# Contactless Sleep Monitoring for Early Detection of Health Deteriorations in Community-Dwelling Older Adults: Exploratory Study

**DOI:** 10.2196/24666

**Published:** 2021-06-11

**Authors:** Narayan Schütz, Hugo Saner, Angela Botros, Bruno Pais, Valérie Santschi, Philipp Buluschek, Daniel Gatica-Perez, Prabitha Urwyler, René M Müri, Tobias Nef

**Affiliations:** 1 Gerontechnology and Rehabilitation Group ARTORG Center for Biomedical Engineering Research University of Bern Bern Switzerland; 2 Department of Cardiology University Hospital Bern University of Bern Bern Switzerland; 3 I.M. Sechenov First Moscow State Medical University Moscow Russian Federation; 4 La Source School of Nursing Sciences HES-SO University of Applied Sciences and Arts of Western Switzerland Lausanne Switzerland; 5 DomoSafety SA Lausanne Switzerland; 6 Idiap Research Institute Martigny Switzerland; 7 École Polytechnique Fédérale de Lausanne Lausanne Switzerland; 8 Department of Neurology University Hospital Bern University of Bern Bern Switzerland

**Keywords:** sleep restlessness, telemonitoring, digital biomarkers, contactless sensing, pervasive computing, home-monitoring, older adults, toss and turns, sleep monitoring, body movements in bed

## Abstract

**Background:**

Population aging is posing multiple social and economic challenges to society. One such challenge is the social and economic burden related to increased health care expenditure caused by early institutionalizations. The use of modern pervasive computing technology makes it possible to continuously monitor the health status of community-dwelling older adults at home. Early detection of health issues through these technologies may allow for reduced treatment costs and initiation of targeted preventive measures leading to better health outcomes. Sleep is a key factor when it comes to overall health and many health issues manifest themselves with associated sleep deteriorations. Sleep quality and sleep disorders such as sleep apnea syndrome have been extensively studied using various wearable devices at home or in the setting of sleep laboratories. However, little research has been conducted evaluating the potential of contactless and continuous sleep monitoring in detecting early signs of health problems in community-dwelling older adults.

**Objective:**

In this work we aim to evaluate which contactlessly measurable sleep parameter is best suited to monitor perceived and actual health status changes in older adults.

**Methods:**

We analyzed real-world longitudinal (up to 1 year) data from 37 community-dwelling older adults including more than 6000 nights of measured sleep. Sleep parameters were recorded by a pressure sensor placed beneath the mattress, and corresponding health status information was acquired through weekly questionnaires and reports by health care personnel. A total of 20 sleep parameters were analyzed, including common sleep metrics such as sleep efficiency, sleep onset delay, and sleep stages but also vital signs in the form of heart and breathing rate as well as movements in bed. Association with self-reported health, evaluated by EuroQol visual analog scale (EQ-VAS) ratings, were quantitatively evaluated using individual linear mixed-effects models. Translation to objective, real-world health incidents was investigated through manual retrospective case-by-case analysis.

**Results:**

Using EQ-VAS rating based self-reported perceived health, we identified body movements in bed—measured by the number toss-and-turn events—as the most predictive sleep parameter (*t* score=–0.435, *P* value [adj]=<.001). Case-by-case analysis further substantiated this finding, showing that increases in number of body movements could often be explained by reported health incidents. Real world incidents included heart failure, hypertension, abdominal tumor, seasonal flu, gastrointestinal problems, and urinary tract infection.

**Conclusions:**

Our results suggest that nightly body movements in bed could potentially be a highly relevant as well as easy to interpret and derive digital biomarker to monitor a wide range of health deteriorations in older adults. As such, it could help in detecting health deteriorations early on and provide timelier, more personalized, and precise treatment options.

## Introduction

In the face of an aging society in many countries, aging in place, often tied to home-care services, has attracted increasing public interest [1,2]. There is a clear preference and economic incentive for people to stay in their known environment [2,3]. Early institutionalization puts a significant additional burden on health care systems that are already struggling to keep up with the ongoing demographic changes [4]. An emerging trend in health care under these circumstances is the advent of pervasive computing technology [5-8]. Available research indicates good acceptance of such technology among older adults if the technology used does not require any interaction (ie, contactless) and is unobtrusive (ie, no voice or video recordings) [9-14]. This makes commonly used wearable devices, often encountered in younger demographics, suboptimal for long-term use with older adults.

Besides detecting acute events, such as falls, stroke, or acute heart failure, a potential application of this technology in older adults could be the detection of early signs of health issues [15]. Toward this goal, it is crucial to know which digital measures of health are useful early indicators of general health issues.

Compared with traditional biological biomarkers, objective health-relevant parameters obtained by means of modern information technology and outside of clinical environments are increasingly termed digital biomarkers [16-18]. Research in this domain is still relatively scarce but promising overall. Rantz et al [15], for instance, showed that using a variety of digital biomarkers they could successfully detect early changes in the health status of older adults. Moreover, they demonstrated that older adults with care enhanced through unobtrusive pervasive computing systems had better overall health outcomes compared with a control group. In the same line of research, Skubic et al [19] identified the most important features for the discrimination between useful and nonuseful health alerts as retrospectively judged by health care professionals. They found the most useful marker, in terms of useful to non-useful ratio, to be bed restlessness events.

Sleep is vital for normal physiologic functioning, with sleep problems becoming more prevalent with age. While some problems are thought to be a result of normal aging processes, many are indicative of underlying medical or psychological disorders or comorbidities [20]. In this context, Adam et al [21] found that community-dwelling older women with higher sleep onset delay (the time it takes to fall asleep) and lower sleep efficiency (fraction of time spent asleep while in bed) were significantly more likely to be placed in a nursing home. It has also been shown that sleep-related problems are an important factor driving institutionalizations among older adults and that they are often poorly recognized by caregivers [21,22]. As a result of people being stationary during sleep, contactless modern sensor technology can be used to measure sleep without device interactions or the necessity to wear and maintain devices likes smartwatches or fitness trackers [23-25]. In addition, sleep is much more comparable than daytime parameters as it generally occurs in the same setting, without many external factors being involved such as visitors, different activities, or different locations.

Considering these factors, previous findings, and the strong link between sleep and health in older adults, it seems that long-term sleep monitoring in older adults could be very useful for early detection and monitoring of health-related problems. However, further research investigating remote monitoring-derived sleep parameters as potential early indicators for general health issues is lacking. Toward this goal, we analyzed real-world longitudinal remote monitoring data from 37 older community-dwelling adults comprising more than 6000 nights worth of in-home sleep recordings and more than 600 matched health reports.

## Methods

### Overview

We performed a data-driven exploratory analysis of sleep data stemming from 37 older adult (aged 70 to 101 years) participants in Switzerland who were monitored for a target duration of at least 1 year, totaling 6686 recorded nights after preprocessing. Analysis aimed to identify sleep parameters that influenced health status. Since this is a hardly quantifiable and often subjective outcome, we used regularly reported perceived health reports as a proxy. To evaluate the effect on real-world health-related events, we further conducted manual case-by-case analysis, validating the most predictive sleep parameter of perceived health by using weekly health reports.

### Participants

The analyzed data stems from 2 cohorts of Swiss seniors (pooled mean age 87 [SD 7] years; sex 67% [30/45] female) where novel computing technologies for aging in place scenarios were evaluated. Participants from both cohorts were recruited to represent naturalistic samples of community-dwelling, alone-living older adults in Switzerland [26,27]. Both studies took place between 2017 and 2018. The inclusion criteria were similar in that they aimed to recruit older adults aged 70 years and older who were living alone in an apartment or house without pets. In cohort 2, participants were additionally required to be followed by a local home-care association. While in cohort 1, only unwillingness to comply with the study protocol was an exclusion criterion, cohort 2 had the following exclusion criteria: (1) severe cognitive impairment making one unable to follow study protocol (clock-drawing score ≥4); (2) skin problems, such as irritations, itching, serious redness; (3) undergoing dialysis; (4) not willing to comply with the study protocol; (5) unable to understand the study aim; or (6) hospitalization planned in a short period of time [26]. The related studies were both conducted based on principles in the Declaration of Helsinki and approved by the Ethics Committee of the canton of Bern and Vaud, Switzerland (KEK-ID: 2016-00406 and CER-VD ID: 2016-00762, respectively). All subjects signed and returned an informed consent before study participation. We included all participants who started in either study, giving us a combined total of 45 participants (not all had matching data as some dropped out early).

### Cohort Differences and Characteristics

In both studies, the same device for sleep recording and the same protocols for interviews and questionnaires were used. There are no differences between the study protocols with regard to the analyzed sleep parameters or health reports. The original studies were conducted by, in part, overlapping researchers but with distinct collaborators and local personnel (same concept, same subset of sensors, same questionnaires and reporting protocol, different technical personnel, different personnel conducting questionnaires and interviews, different personnel responsible for recruitment, different regions in Switzerland). Participant characteristics are shown in Table 1. Statistical significances of the respective baseline characteristics were evaluated using 2-sample, 2-sided *t* tests.

**Table 1:** Participant and questionnaire characteristics.

Characteristic	Cohort 1 (n=24)	Cohort 2 (n=21)	Cohort differences, *t* score (*P* value)	Pooled (n=45)
Age (years), mean SD	88 (7)	86 (7)	–0.76 (.50)	87 (7)
Sex, female, n (%)	19 (79)	11 (52)	1.94 (.06)	30 (67)
EQ-VAS^a^, mean (SD)	78 (13)	72 (15)	1.35 (.19)	75 (14)
Nights measured, n (%)	4806 (87)	1880 (61)	—^b^	6686 (78)
Nights measured, mean (SD)	200 (104)	104 (97)	3.03 (.004)	159 (111)
Health reports, n (%)	963 (—)	803 (—)	—	1766 (—)
Health reports, mean (SD)	38 (10)	38 (24)	0.03 (.98)	38 (18)
Health reports, matched, n (%)	417 (43)	234 (29)	—	651 (37)
Health reports, matched, mean (SD)	18 (9)	15 (17)	0.56 (.58)	17 (13)

^a^EQ-VAS: EuroQol visual analog scale.

^b^Not applicable.

### Ground Truth Data Acquisition

Perceived health and health-relevant events were recorded on a weekly basis by means of short interviews and questionnaires. These weekly reports were gathered as part of home visits and were mandatory for study participants to answer. To evaluate the participants’ perception of health status, we used the well-validated EuroQol EQ-5D-3L health-related quality of life instrument.

The EQ-5D-3L is a well-established instrument to measure quality of life and has been shown to be consistently associated with mortality and hospitalization in older adults [28]. For simplicity reasons, we only used the EQ-VAS part of the EQ-5D-3L as it has been shown to exhibit stronger associations with number of hospitalizations and long-term mortality in older adults in comparison to the EQ-5D-3L index and classes of problems [28,29]. The EQ-VAS asks participants to describe their health state on a visual analog scale that ranges from 0 to 100 [30].

### Sensor Data Acquisition

Sleep was monitored with a contactless, ferroelectret bed sensor (EMFIT QS, Emfit Ltd), which was placed under a person’s mattress. The sensor comprises a thin quasi-piezoelectric film that translates thickness differentials into electrical charges [31]. The acquired sleep parameters were extracted by proprietary algorithms from the device manufacturer. Literature supports the accuracy of corresponding algorithms. It has notably been shown that the algorithms used with the device are capable of measuring heart rate and respiration rate with good accuracy [31]. Moreover, sleep staging algorithms of Emfit-based sensors have shown good agreement when compared with polysomnography-based ratings [32,33]. The devices were connected to the internet via specifically set up Wi-Fi hotspots. Sleep data recorded at the participants’ homes was sent to a cloud in real time and automatically acquired for analysis.

### Data Processing

**Figure 1:**
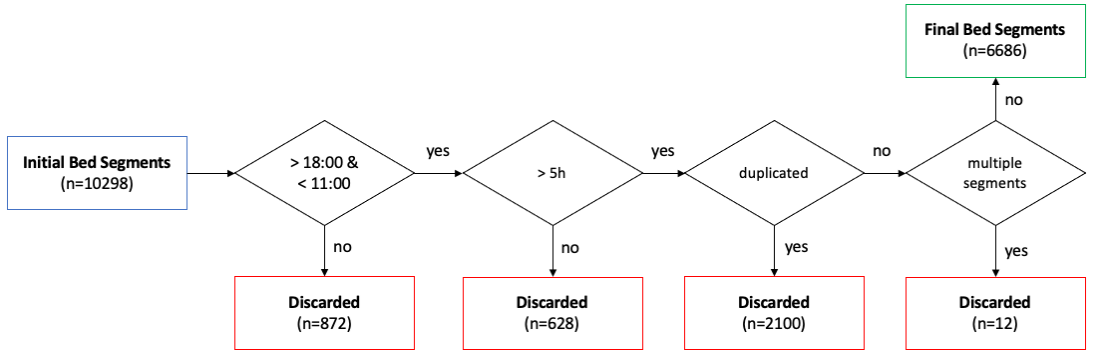
Preprocessing flowchart.

The acquired sleep segments were first filtered by their recording start and stop time (after 6 PM and before 11 AM) to exclude potential daytime naps from being included. Furthermore, only segments with a total duration of more than 5 hours were included, and duplicated segments were removed. Last, if multiple segments were recorded for a single night slot, the longest recording was used. Segments were defined as continuous bed activity with no more than 2 hours of out-of-bed time in between. A graphical overview is given in Figure 1.

Before modeling, all sleep data was standardized to zero mean and unit variance. To assess the relationship of sleep data with EQ-VAS ratings, sleep parameters of the night proceeding the questionnaire completion were matched with the respective EQ-VAS ratings. This resulted in a total of 651 matching data points across 37 participants. Every participant with at least one matching data point was included in the analysis.

### Data Loss

Since the EMFIT sensors had to be connected to local Wi-Fi, numerous nights were lost due to connectivity issues with the mobile devices providing the hotspots (this includes loss of Wi-Fi connection, loss of 3G connectivity, network provider issues, and server issues). These problems were amplified by the logistical difficulty of fixing technical issues with remote monitoring in a timely manner. To reduce the risk of a potential bias as a result of missing nights, we evaluated the relationship between the percentage of missing matched data points and EQ-VAS ratings by means of the Spearman correlation coefficient of the 2 variables. To check for a potential bias introduced by the exclusion of participants without matching data points (primarily those who dropped out earlier), we performed a 2-sample, 2-sided *t* test to test for a significant difference in the means of EQ-VAS ratings between the participants included and the ones excluded as a result of not having matching data for this analysis.

### Sleep Parameters

The analyzed sleep parameters include commonly measured quality-of-sleep metrics such as sleep efficiency, sleep latency, number of awakenings, rapid eye movement (REM) sleep, deep sleep, and light sleep [34]. Additionally, body movements, further referred to as the number of toss-and-turn events, as the parameter is named by the manufacturer, were analyzed. What counts as body movement is not exactly disclosed by the manufacturer but can be broadly described as a binary discretization (dichotomization) of the activity signal over short-time epochs of a few seconds. This is conceptually similar to what is being done when measuring activity counts with wearable accelerometers. A full overview of the included sleep parameters is given in Table 2 and Multimedia Appendix 1. All parameters are derived from proprietary algorithms from the manufacturer.

**Table 2:** Sleep parameters.

Parameter	Value, mean (SD)
Duration total (first to last recorded event in bed, sec)	31,823 (5776)
Duration in bed (sec)	30,788 (5607)
Average heart rate (beats per minute)	61.83 (6.32)
Average respiration rate (breaths per minute)	14.60 (2.78)
Number bed exits (count)	3.01 (2.50)
Number toss-and-turn events (larger movements, count)	57.62 (61.17)
Duration asleep (sec)	26,730 (5474)
Duration in REM^a^ (sec)	6546 (2059)
Duration in light sleep (sec)	15,575 (3227)
Duration in deep sleep (sec)	4608 (1665)
Duration awake (sec)	4959 (2041)
Sleep onset delay (sec)	1446 (763)
Duration out of bed (sec)	1034 (914)
Heart rate variability high frequency band, 0.15-0.40 Hz (normalized power spectral density)	56.00 (11.35)
Heart rate variability low frequency band, 0.04-0.15 Hz (normalized power spectral density)	43.62 (11.13)
Number awakenings (count)	2.22 (1.44)
Percentage in deep sleep (%)	0.14 (0.04)
Percentage in REM sleep (%)	0.21 (0.05)
Percentage in light sleep (%)	0.49 (0.06)
Sleep efficiency (time asleep from total duration, %)	0.84 (0.07)

^a^REM: rapid eye movement.

### Ranking of Relevant Sleep Parameters

To quantify the importance of individual sleep parameters on weekly reported EQ-VAS ratings, we used linear mixed-effects models (LMMs) with a random intercept per participant. For each sleep parameter, one model with EQ-VAS rating as response variable, corrected for age and sex, was fit. The rationale for using this type of model is based on the structure of the data: namely, multiple measures (with an uneven number of data points collected) per participant. Eventually, sleep parameters were ranked by the absolute value of their *t* scores. Reported *P* values were calculated on the basis of an implementation introduced by Kuznetsova et al [35]. We additionally report the respective *P* values when corrected for multiple comparisons, using the Bonferroni-Holm method. To provide insights into how the highest ranked predictor is associated with the remaining sleep parameters, individual Spearman correlation coefficients (*r*) were calculated. For all reported statistical hypothesis tests, we set a significance level of *α*=.05.

### Case-by-Case Analysis of Toss-and-Turn Metric

While the above-mentioned ranking helps to identify the most relevant metric with regard to self-reported health, it does not necessarily imply a relevant effect on actual health. To check for an effect of the most relevant metric with regard to actual health, we performed manual, retrospective case-by-case analysis to investigate whether visually perceivable trends could be explained by health reports. For each manually identified trend (based on 14-day moving average) or point anomaly with regard to toss-and-turn time series, we analyzed the respective health reports around the same time to verify whether any health-relevant events that could explain the observations were reported.

### Quantifying Anomalous Sleep Restlessness Evolutions

While manual inspection of the toss-and-turn metric may be possible in some settings, it is time-consuming and might thus not be practical. A relatively straightforward way to quantify structural changes in toss-and-turn counts, beyond just percentual changes, is to view them as the result of a Poisson process. In the case where the toss-and-turn number is approximately stable, this process can be seen as stationary (homogenous). In the presence of external factors, such as potential health changes, the toss-and-turn counts can be thought of as the result of a nonstationary Poisson process. To differentiate between the two, we assume the former and calculate the maximum likelihood estimate for the rate parameter λ*_TnT_* to calculate the chi-square statistic 
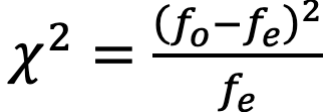
 of the observed *f_o_* and expected count frequencies *f_e_*. In case the process is nonstationary, the chi-square statistic will become increasingly large and allows for a threshold to be set for automated notifications. To analyze the association of this approach with perceived health, we performed calculations based on weekly segmented toss-and-turn data and event counts in 30-minute time intervals. EQ-VAS measures for the same week were matched and averaged.

### Software Used

Data acquisition, preprocessing, and sleep parameter extraction were performed using the Python (Python Software Foundation) programming language. Statistical tests and LMMs were computed using the R statistical programming language (R Foundation for Statistical Computing) with package lme4 version 1.1-21 [36].

## Results

### Ranking of Relevant Sleep Parameters

**Figure 2:**
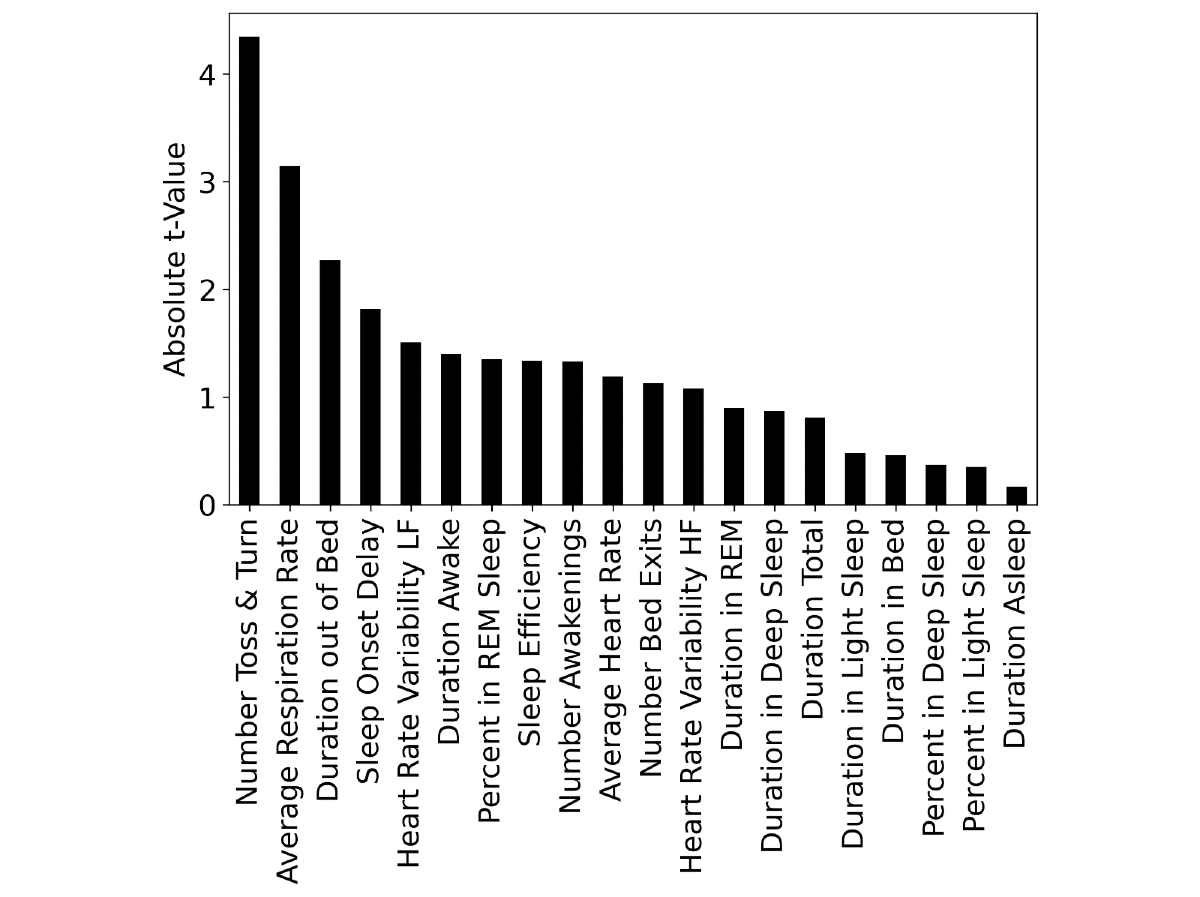
Ranking of self-rated perceived health based on individual linear mixed effects models.

**Figure 3:**
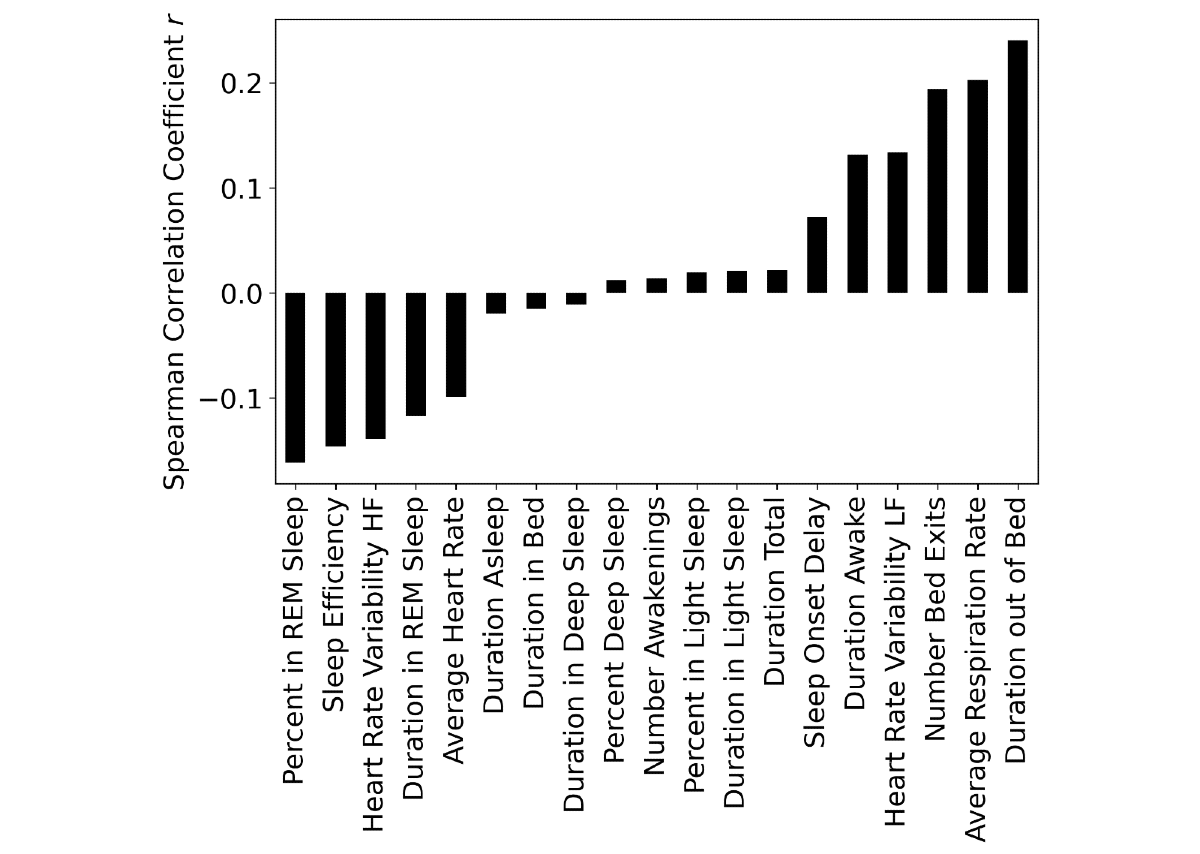
Relationship between number of toss-and-turns and the remaining sleep parameters.

We found the strongest predictor of perceived health to be the total number of toss-and-turn events per night (*t* score=–0.435, *P*<.001, *P* [adj]<.001). The only other predictor to remain significant after correcting for multiple comparison was the average nightly respiration rate (*t* score=–3.148, *P*=.002, *P* [adj]=.032). Both variables showed a negative association with EQ-VAS ratings. The full ranking is visualized in Figure 2, and all results are presented in Table 3. Overall associations between nightly toss-and-turn counts and the remaining sleep parameters based on Spearman correlation coefficients are shown in Figure 3. The strongest positive association was with the duration spent out of bed (*r*=.24) and the strongest negative one was found to be with the percentage spent in REM sleep (*r*=–.16). All associations were statistically significant, primarily as a result of the large number of 6686 data points.

**Table 3:** Association of EuroQol visual analog scale ratings with sleep parameters based on mixed effects models.

Parameter	Estimate	*t* score	*P* value	*P* value (adjusted)
Number toss-and-turn events	–2.48	–4.35	<.001	<.001
Average respiration rate	–1.81	–3.15	.002	.03
Average heart rate	–0.73	–1.19	.23	>.99
Duration total	–0.38	–0.81	.42	>.99
Duration in bed	–0.21	–0.46	.65	>.99
Number bed exits	–0.55	–1.13	.26	>.99
Duration asleep	–0.08	–0.17	.86	>.99
Duration in REM^a^	0.36	0.90	.36	>.99
Duration in light sleep	–0.20	–0.48	.63	>.99
Duration in deep sleep	–0.30	–0.87	.39	>.99
Duration awake	–0.57	–1.40	.16	>.99
Sleep onset delay	–0.61	–1.82	.07	>.99
Duration out of bed	–1.03	–2.27	.02	.42
Heart rate variability high frequency band	–0.44	–1.08	.28	>.99
Heart rate variability low frequency band	0.61	1.51	.13	>.99
Number awakenings	0.57	1.33	.19	>.99
Percentage in deep sleep	–0.12	–0.37	.71	>.99
Percentage in REM sleep	0.49	1.35	.18	>.99
Percentage in light sleep	0.13	0.35	.73	>.99
Sleep efficiency	0.54	1.34	.18	>.99

^a^REM: rapid eye movement.

### Cohort Differences and Data Loss

Regarding potential biases in the above presented results, we found that the difference in participant characteristics of the analyzed cohorts was mostly insignificant (supporting the null hypothesis that the means of the 2 cohorts are equal) with the exception of the number of measured nights per person (*t* score=3.03, *P*=.004); more details can be found in Table 1. We further found a nonsignificant and very weak correlation between the percentage of missing nights and reported EQ-VAS ratings per participant (*r*=–.10, *P*=.54, n=37). Regarding participants who were not included in the analysis as a result of not having matching nights measured, we found no significant difference in the means of EQ-VAS ratings (*t* score=–0.44, *P*=.66).

### Case-by-Case Analysis

Results from qualitative case-by-case analysis, where trends and point anomalies with regard to the number of toss-and-turns per night were manually analyzed, are summarized in Table 4. The analysis showed that more than half (7/13, 54%) of abnormally looking toss-and-turn patterns could be related to reports of health-relevant events. Cases with a particularly high increase of more than 200 toss-and-turns per night (3 in total) were all accompanied by medically relevant events, of which 2 were severe and led to hospitalizations and death, respectively. Reported health incidents with visible change in toss-and-turns include heart failure, hypertension, abdominal tumor, seasonal flu, gastrointestinal problems, and urinary tract infection.

**Figure 4:**
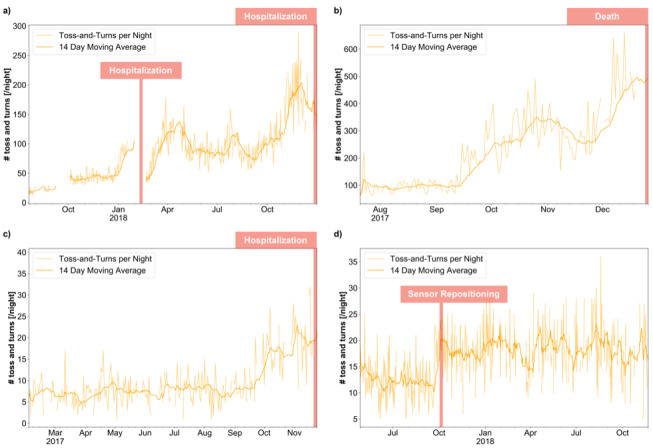
Evolutions of nightly toss-and-turn counts related to reported events.

Four examples are displayed in Figure 4, where Figure 4A depicts the case of a participant who was hospitalized twice due to heart failure. Both hospitalizations were preceded by increases in the number of toss-and-turn events. It should be noted how the toss-and-turn numbers stabilized for a short time period after the first hospitalization and the resulting compensation. Figure 4B shows the number of nightly toss-and-turn events of a participant experiencing a rapid decline in health, later diagnosed as a large abdominal tumor and worsening heart failure, eventually leading to the participant’s death. It is apparent how the number of toss-and-turn events per night increased strongly, with a small decrease prior to a final increase. Figure 4C shows the case of a participant who was hospitalized and subsequently institutionalized as a result of worsening hypertension problems. About 2 months prior to institutionalization, the number of toss-and-turn events started to increase markedly. Finally, in Figure 4D, a participant without any health issues is shown. The only visible (abrupt) signal change was due to a reported sensor repositioning.

**Table 4:** Summary of qualitative analysis of abnormal toss-and-turn patterns.

Identified Anomalous Patterns	Total cases, n	Cases with plausible explanation, n (%)
Trends (≤200 toss-and-turns)	13	7 (54)
Trends (>200 toss-and-turns)	3	3 (100)
Abnormal local peaks	20	13 (65)

### Quantifying Anomalous Sleep Restlessness Evolutions

**Figure 5:**
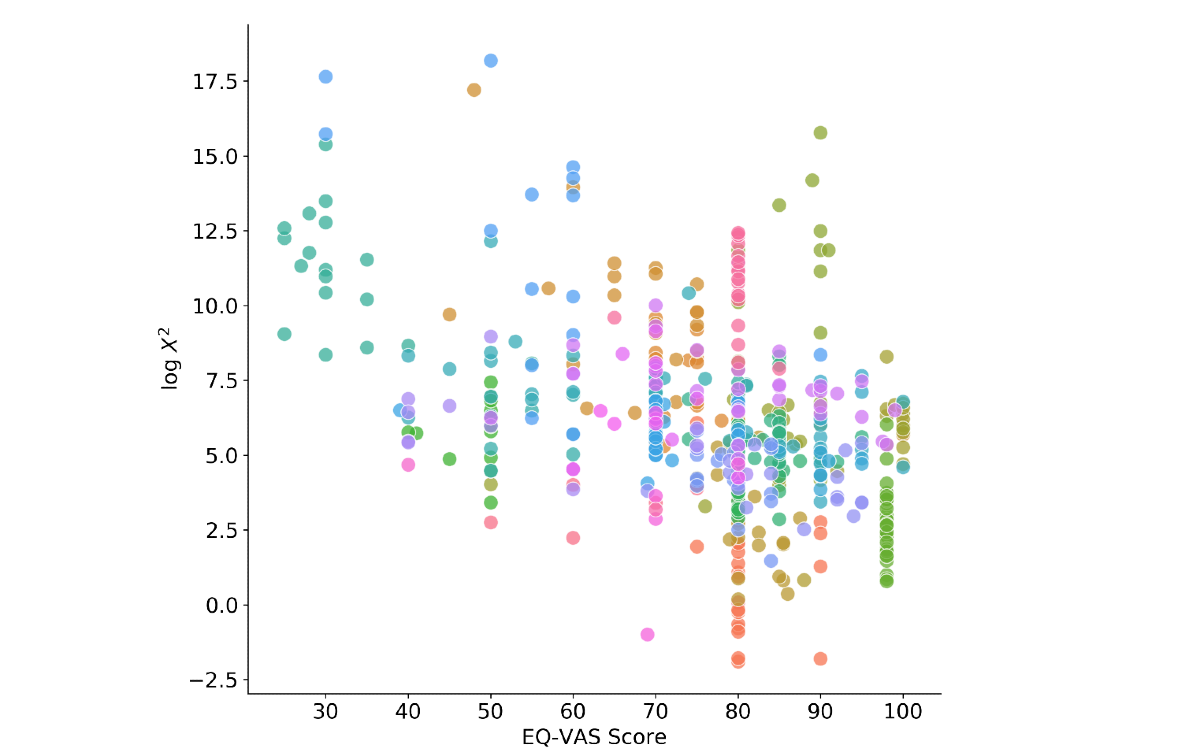
Relationship between chi-square statistics and EuroQol visual analog scale (EQ-VAS) ratings.

As can be seen in Figure 5, the log transformed chi-square statistics of weekly time intervals and matched EQ-VAS ratings show a negative association (*r*=–.42, *P*<.001, n=588) such that a higher chi-square indicates lower self-reported health. However, exact cutoff values that could be used to trigger alarms are difficult to find, since we do not have an absolute definition for relevant health status changes. We found that, in the analyzed population, weekly chi-square values greater than 1000 were mostly related to health changes and other sensor anomalies like displacements.

## Discussion

### Principal Findings

We retrospectively analyzed real-world, long-term remote monitoring sleep data from two studies in Switzerland where older adults’ homes were equipped with modern pervasive computing systems and followed weekly by nursing staff. The goal of this explorative analysis was to find which unobtrusively measurable sleep parameter was most indicative of general health deteriorations in community-dwelling older adults. Among 20 daily extracted sleep parameters, the number of toss-and-turn events per night, which can be thought of as the amount of body movements, was found to exhibit the strongest association with weekly EQ-VAS based self-rated health ratings. It should be mentioned that this association is likely not only a subjective momentary feeling since the EQ-VAS is known to be strongly related to all-cause mortality and hospitalizations in older adults [28,29]. In accordance with this relationship, we found that the toss-and-turn parameter often exhibits very apparent changes that we could relate to reported health incidents. As such, all cases leading to more than 200 toss-and-turn events per night were related to reported health-relevant incidents. But smaller trends could also be explained by events mentioned in health reports in at least half of the cases. Encountered incidents include heart failure, hypertension, abdominal tumor, seasonal flu, gastrointestinal problems, and urinary tract infection.

Since long-term home-monitoring in older adults is a new field with very few research groups actually performing real-world studies, there is not much research to relate these findings to. However, one of the pioneering groups in this field reported a surprisingly similar finding. In 2015, Skubic et al [19] found sleep restlessness events measured by pneumatic transducers on top of the mattress to be the variable resulting in the highest ratio of good to poor health alerts in a population of older adults. They assessed a variety of parameters, including activity patterns in different rooms but also discretized heart and breathing rates during sleep. We want to point out that what they called bed restlessness events is conceptually the same as the body movements we call toss-and-turns. In the end, this is a question of nomenclature. While further investigation is certainly necessary, it is unlikely that the similarities in these two independent studies have occurred by chance. The exact reasons as to why body movements exhibit this association with a variety of health deteriorations remains to be elucidated. In theory, most of the involved health incidents are accompanied by inflammation. One potential mechanism could thus be related to immune system–modulated changes as a response to inflammatory processes [37]. Beyond inflammation, it is known that psychosocial stress can also have an impact on various sleep metrics like the number of awakenings or sleep efficiency [38]. Whatever the exact underlying causes, it is not a stretch to assume that increases in toss-and-turns come with more fragmented sleep, more awakenings, and more time spent awake in bed. This is also supported by the positive correlation of toss-and-turns with bed exits, time out of bed, number of awakenings or time spent awake. Additionally, it is known that older adults are at particular risk of being awoken by body movements during sleep – potentially further increasing the relevance of nightly toss-and-turns in this demographic [39].

An interesting aspect of these findings is that well-known sleep quality metrics like sleep duration and sleep efficiency are seemingly less relevant than body movements when it comes to an overall association with perceived health in older adults. Correlation analysis between the toss-and-turn values and those metrics further shows weak overall correlations (see Figure 3) and thus suggests that toss-and-turns actually capture different components of sleep quality behavior.

When it comes to detecting relevant changes in toss-and-turns, interpreting the generation of toss-and-turn events as a Poisson process makes it straightforward to detect even smaller changes in a principled manner and allows for potentially triggering alarms at predefined threshold values. This provides a way of quantifying changes that is likely less prone to a specific implementation of an algorithm that discretizes movements in bed into individual (toss-and-turn) events.

The number of toss-and-turns per night is a sleep parameter that is fairly simple to measure unobtrusively, thus without any interactions or privacy-compromising measurement modalities. Nightly body movements in bed, as quantified by the number of toss-and-turns, could thus potentially be used as a convenient digital biomarker for early detection and monitoring of a wide range of health deteriorations in older adults, including potentially life-threatening conditions like heart failure or certain types of cancer. Early detection in many cases could help to involve medical professionals in early stages of disease and provide more timely treatment options and preventive measures [15,40]. In addition, having a responsive digital biomarker in such cases might also allow for monitoring of the effectiveness of treatments, indicating when a specific treatment plan is failing and needs adjustment. A real-world example of this is the case of heart failure as shown in Figure 4A, where decompensation in the hospital was showing initial relief but, as is well visible by the toss-and-turns, was only temporary and reclined shortly after hospital discharge. This deterioration necessitated further adjustment in medication. The need for such an adjustment might have been detectable earlier by monitoring markers like toss-and-turns proactively.

Given the relationship with EQ-VAS ratings, evidence from real health events, and the similar finding from Skubic et al [19], we find nightly body movements in older adults to be a very promising digital biomarker that needs further investigation. Ideally, future studies should compare against controlled polysomnography measurements and potentially also different devices. Finally, larger datasets in this regard could also allow for a statistically sound evaluation of the effect of sleep parameter combinations.

### Limitations

Working in true remote-monitoring settings comes with downsides and very time-consuming logistics. As such, we had to deal with a nonnegligible number of missing nights, mainly due to connectivity issues with wireless networks. While this factor is most likely independent from participants and thus should not introduce a bias (as supported by the nonexistent association of EQ-VAS scores and missing nights) with respect to our results, it highlights practical problems with this specific setup used to collect the presented data. It should be noted that many of the issues we experienced can and will be solved eventually. Besides technical limitations, there are a few major shortcomings of the presented work.

First, while we did analyze a large number of nights, 37 participants is still a very limited sample size and we are thus not able to provide general information on which kind of health issues can be detected using the toss-and-turn metric.

Second, our analysis might be somewhat biased toward the proprietary algorithms of the device manufacturer. This should not invalidate findings but could mean that difficult-to-measure parameters like heart rate variability or exact sleep stages are exceedingly noisy and might in reality be more important than what we reported, especially as the reliability of such algorithms may deteriorate in presence of more movement.

Third, we did not have access to information about movement-related sleep disorders such as restless leg syndrome, which likely influence the toss-and-turn metric. It should, however, be noted that this would likely not affect the detection of relative changes, which we consider to be the most relevant.

Last, the pooling of the two studies might be a concern. However, apart from the number of missing nights, participant baseline characteristics showed no significant differences and the study protocols with regard to the results shown were the same. The difference in missing nights is explained by different hotspot technologies used and distinct technical personnel being responsible for maintenance. As such, this limitation should be considered but might not really have influenced our results.

### Conclusion

In this study, we evaluated which contactlessly measurable sleep parameter exhibits the strongest association with EQ-VAS ratings in older adults and manually analyzed real-world health events with respect to the parameter with the strongest association. For this we used long-term sleep data acquired with contactless bed sensors in the homes of older community-dwelling adults. We found body movements in bed, quantified by the number of toss-and-turn events, to be the most predictive sleep parameter for EQ-VAS based perceived health ratings among 20 potential sleep parameters. Supporting this finding, increases in toss-and-turn events turned out to often be a precursor of reported real-world health incidents. Furthermore, these results are in accordance with an independent previous finding in literature. Monitoring body movements in bed could thus serve as an interesting and relatively easy to acquire and interpret digital biomarker, allowing health care professionals to proactively screen for and monitor early signs of numerous health deteriorations in older adults. While further evidence from larger, more targeted, studies will be necessary, the potential of such a digital biomarker to be used as digital care support measure might be significant and should be further investigated.

## Appendix

Multimedia Appendix 1 Boxplots of measured sleep parameters for all participants.

## Abbreviations

EQ-5D-3L: EuroQol health-related quality of life instrument
EQ-VAS: EuroQol visual analog scale
LMM: linear mixed-effects model
REM: rapid eye movement
