# Supplementary material for: Contactless Sleep Monitoring for Early Detection of Health Deteriorations in Community-Dwelling Older Adults: Exploratory Study
Source: JMIR Mhealth Uhealth. 2021 Jun 11;9(6):e24666. doi: 10.2196/24666 (PMC8235297; doi:10.2196/24666)
Supplement: Multimedia Appendix 1 [file mhealth_v9i6e24666_app1.pdf]

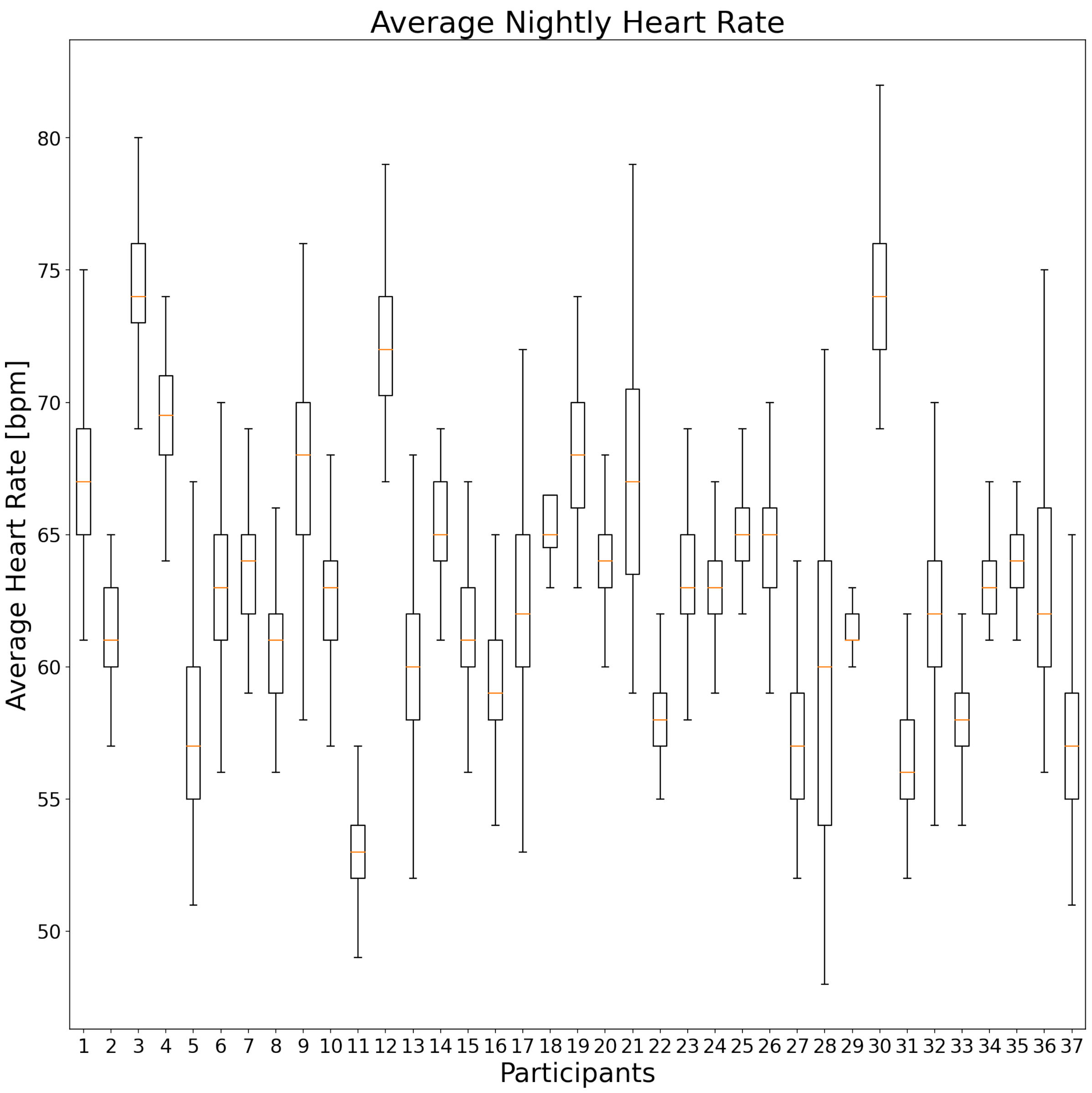

Average Nightly Heart Rate
Average Heart Rate [bpm]
Participants
80
75
70
65
60
55
50
1 2 3 4 5 6 7 8 9 10 11 12 13 14 15 16 17 18 19 20 21 22 23 24 25 26 27 28 29 30 31 32 33 34 35 36 37

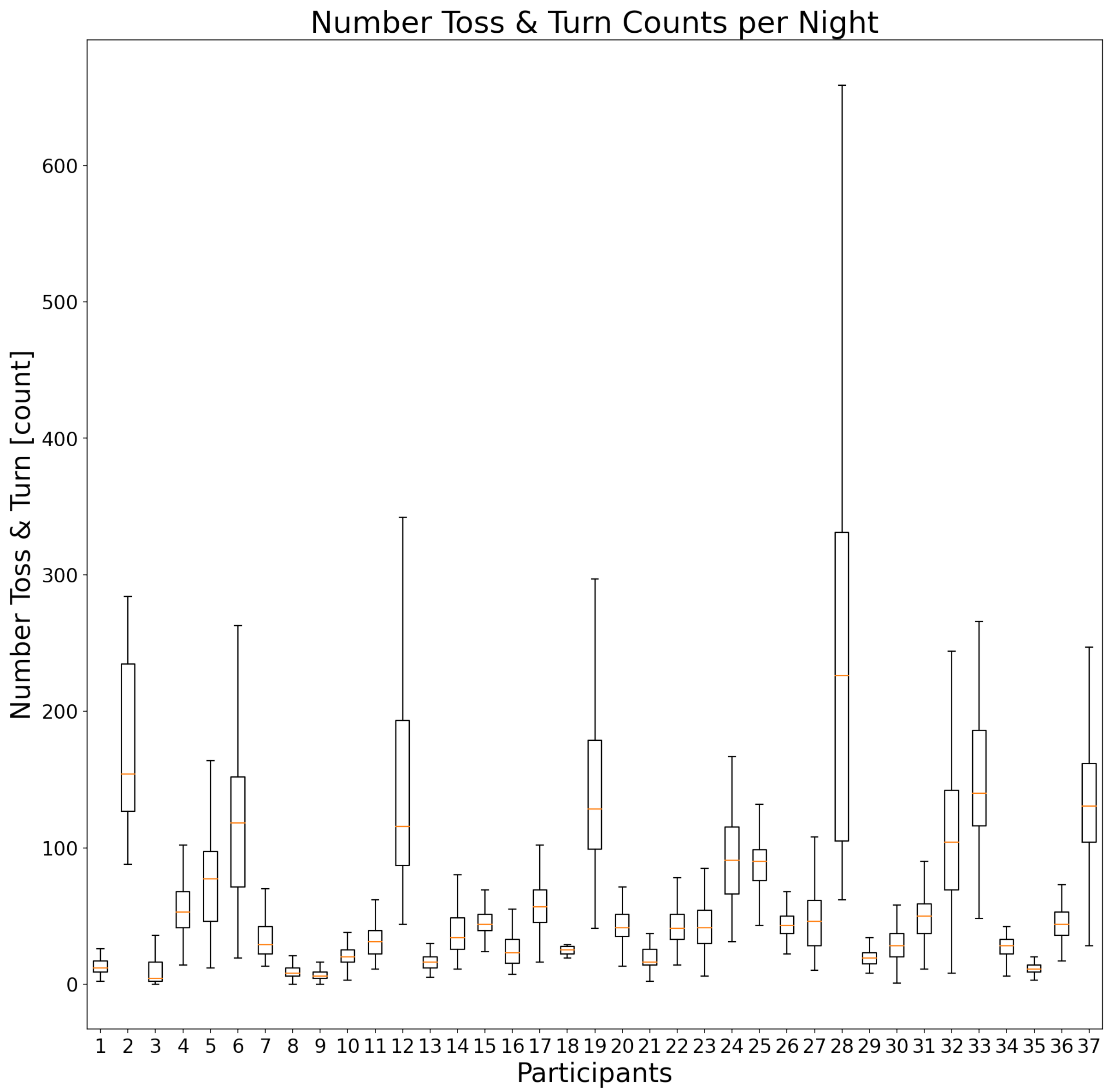

Number Toss & Turn Counts per Night
Number Toss & Turn [count]
0
100
200
300
400
500
600
1 2 3 4 5 6 7 8 9 10 11 12 13 14 15 16 17 18 19 20 21 22 23 24 25 26 27 28 29 30 31 32 33 34 35 36 37
Participants

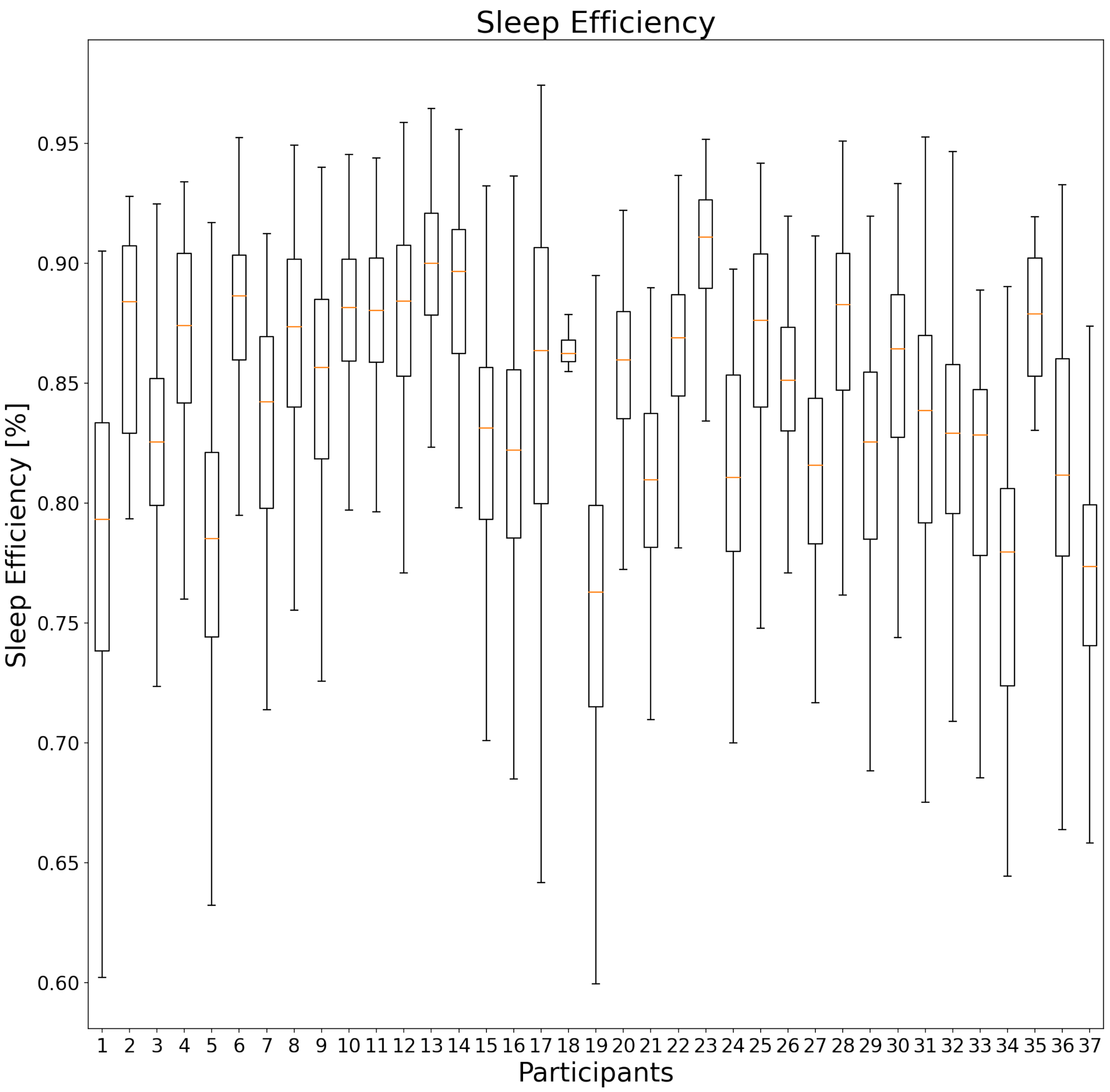
Sleep Efficiency
Sleep Efficiency [%]
0.95
0.90
0.85
0.80
0.75
0.70
0.65
0.60
1 2 3 4 5 6 7 8 9 10 11 12 13 14 15 16 17 18 19 20 21 22 23 24 25 26 27 28 29 30 31 32 33 34 35 36 37
Participants

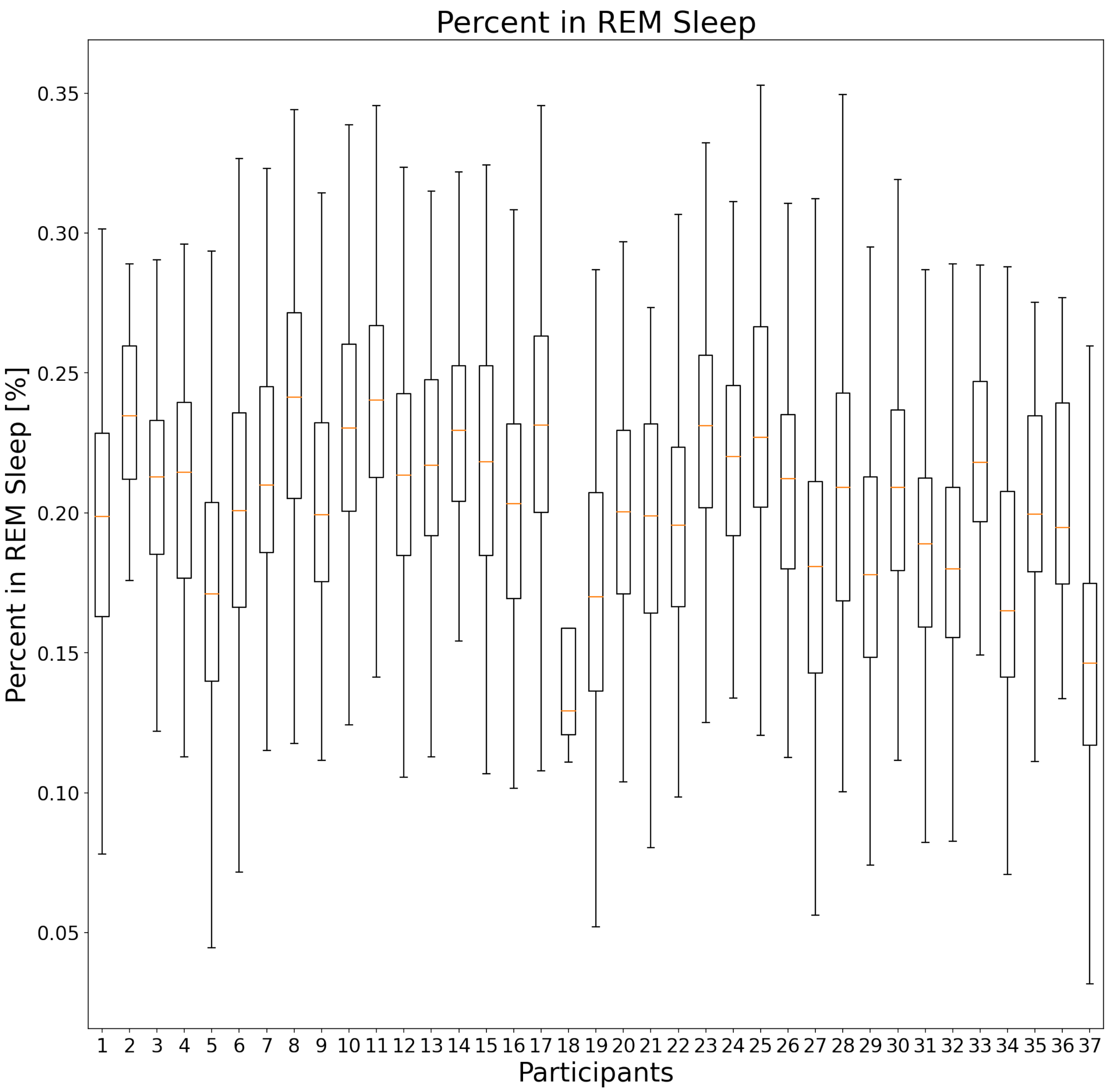

Percent in REM Sleep
Percent in REM Sleep [%]
0.05
0.10
0.15
0.20
0.25
0.30
0.35
1 2 3 4 5 6 7 8 9 10 11 12 13 14 15 16 17 18 19 20 21 22 23 24 25 26 27 28 29 30 31 32 33 34 35 36 37
Participants

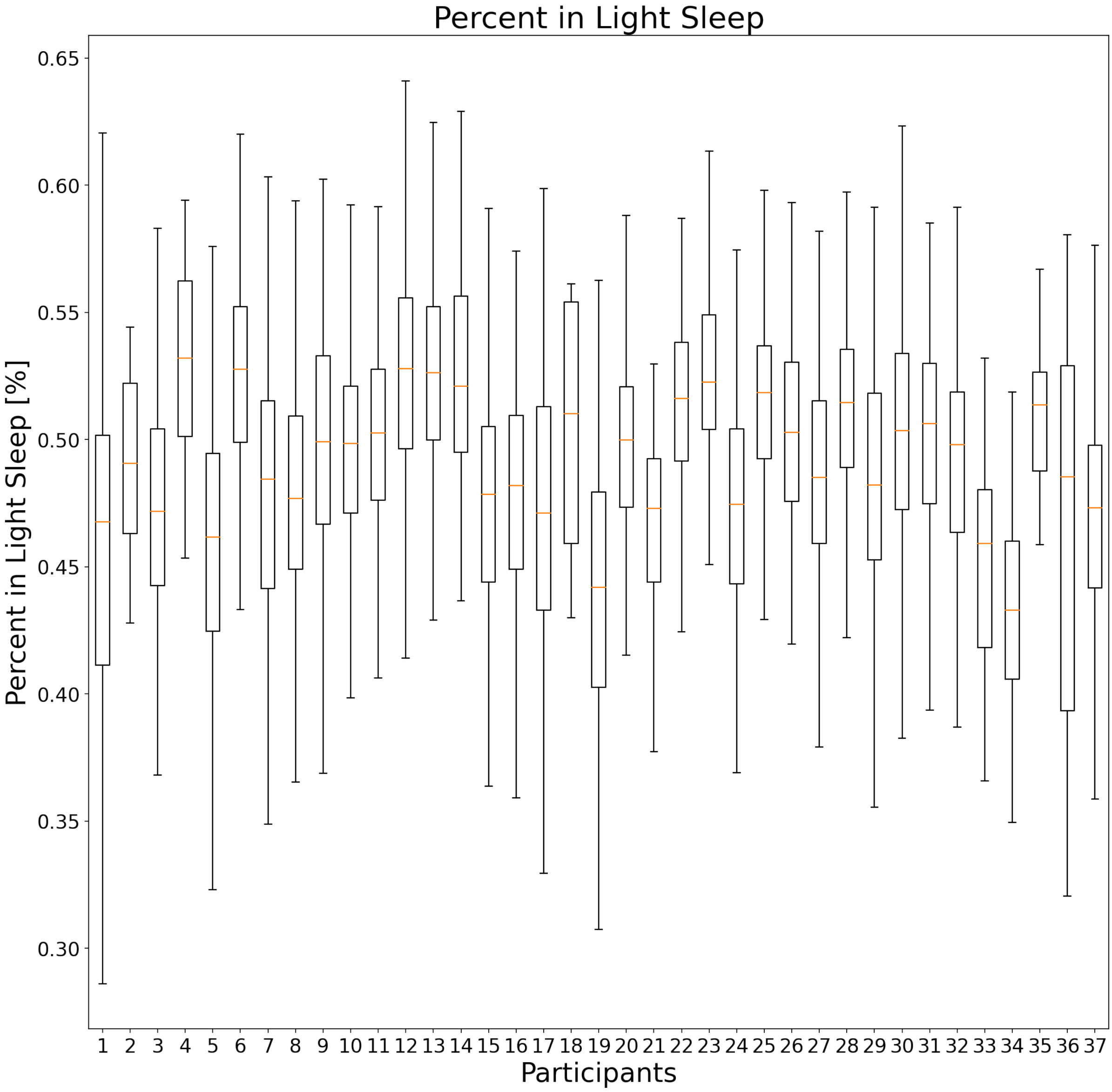

Percent in Light Sleep
Percent in Light Sleep [%]
Participants
0.65
0.60
0.55
0.50
0.45
0.40
0.35
0.30
1 2 3 4 5 6 7 8 9 10 11 12 13 14 15 16 17 18 19 20 21 22 23 24 25 26 27 28 29 30 31 32 33 34 35 36 37

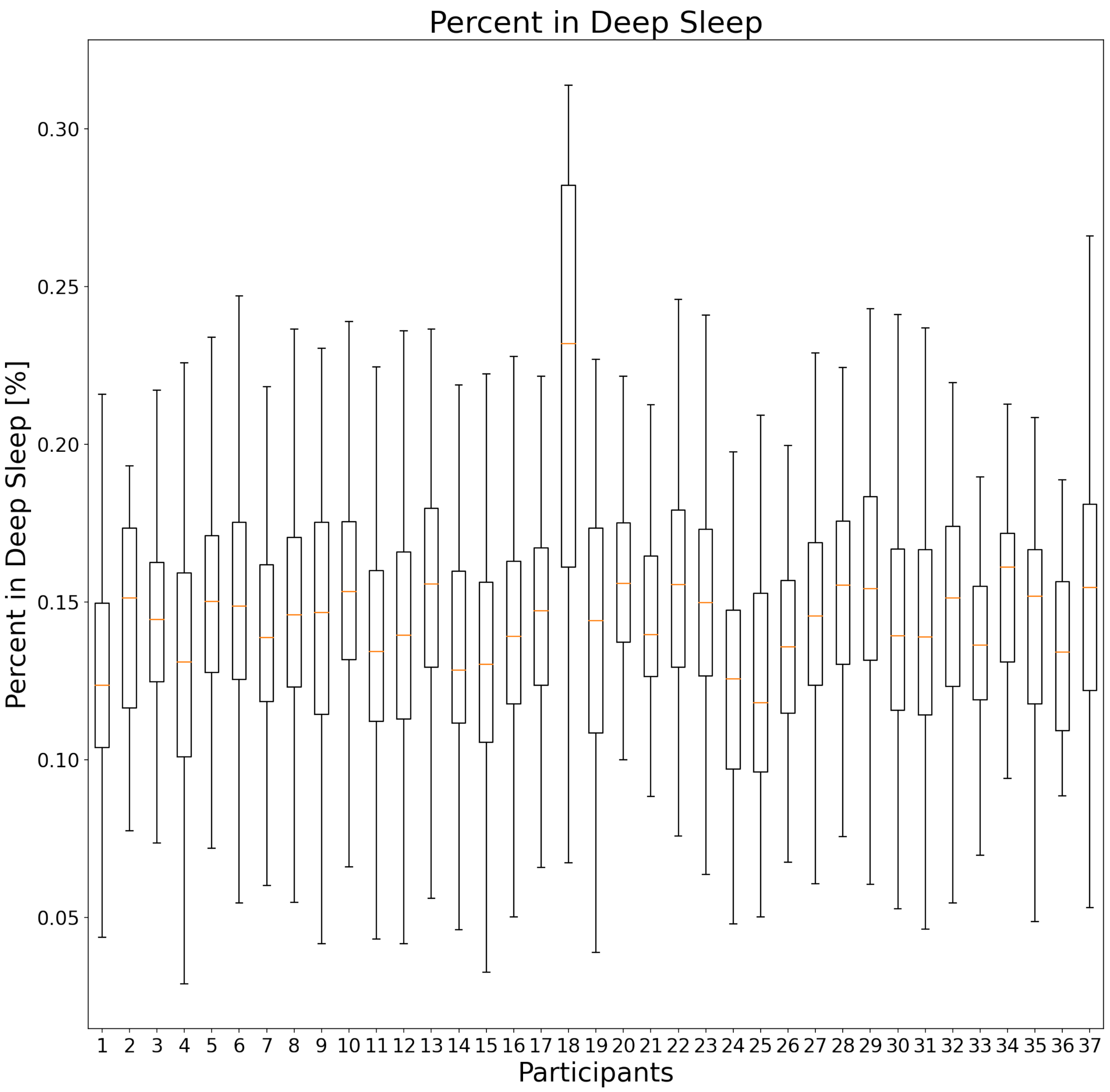
Percent in Deep Sleep
Percent in Deep Sleep [%]
0.30
0.25
0.20
0.15
0.10
0.05
1 2 3 4 5 6 7 8 9 10 11 12 13 14 15 16 17 18 19 20 21 22 23 24 25 26 27 28 29 30 31 32 33 34 35 36 37
Participants

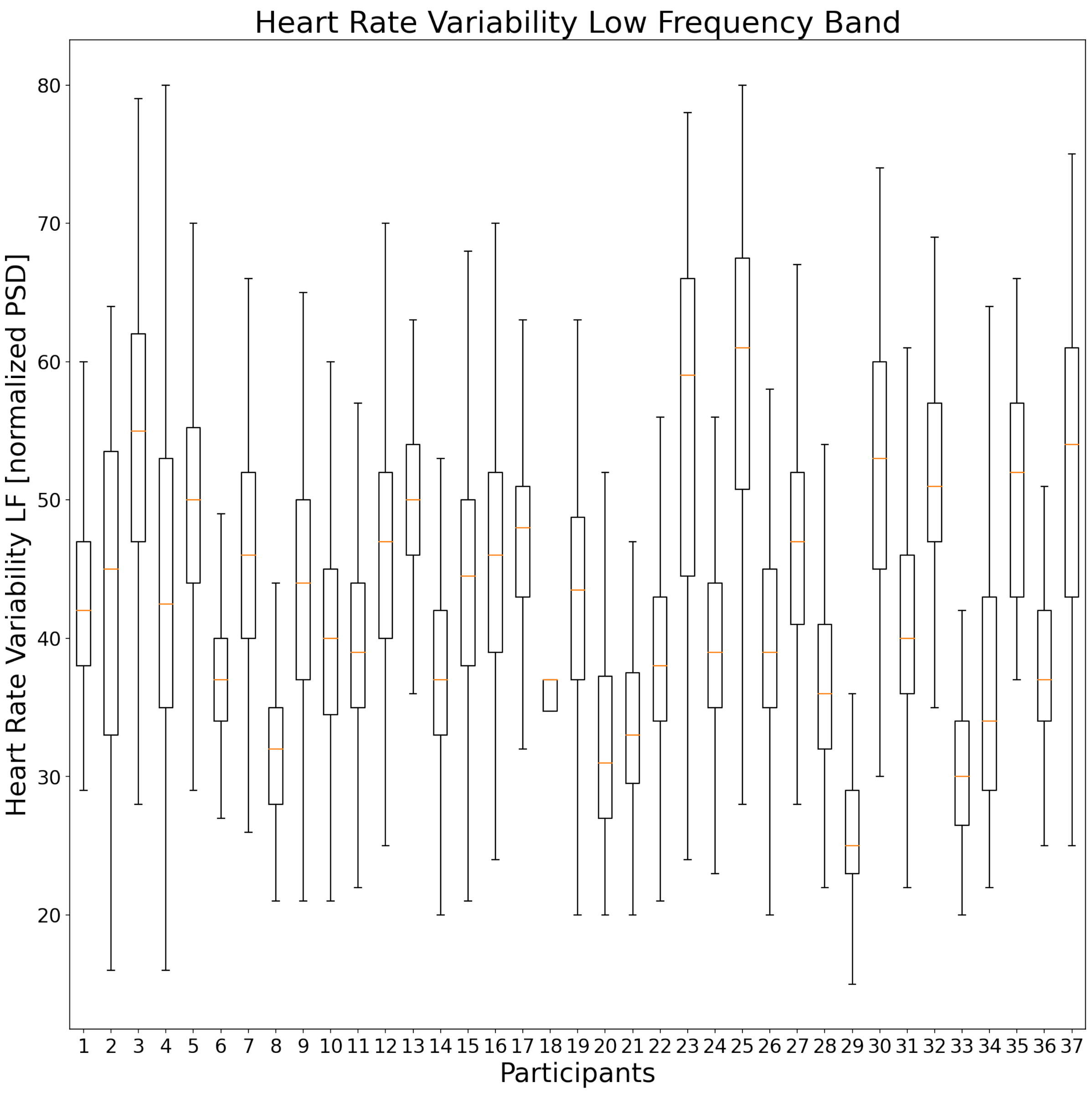

Heart Rate Variability Low Frequency Band
Heart Rate Variability LF [normalized PSD]
20
30
40
50
60
70
80
1 2 3 4 5 6 7 8 9 10 11 12 13 14 15 16 17 18 19 20 21 22 23 24 25 26 27 28 29 30 31 32 33 34 35 36 37
Participants

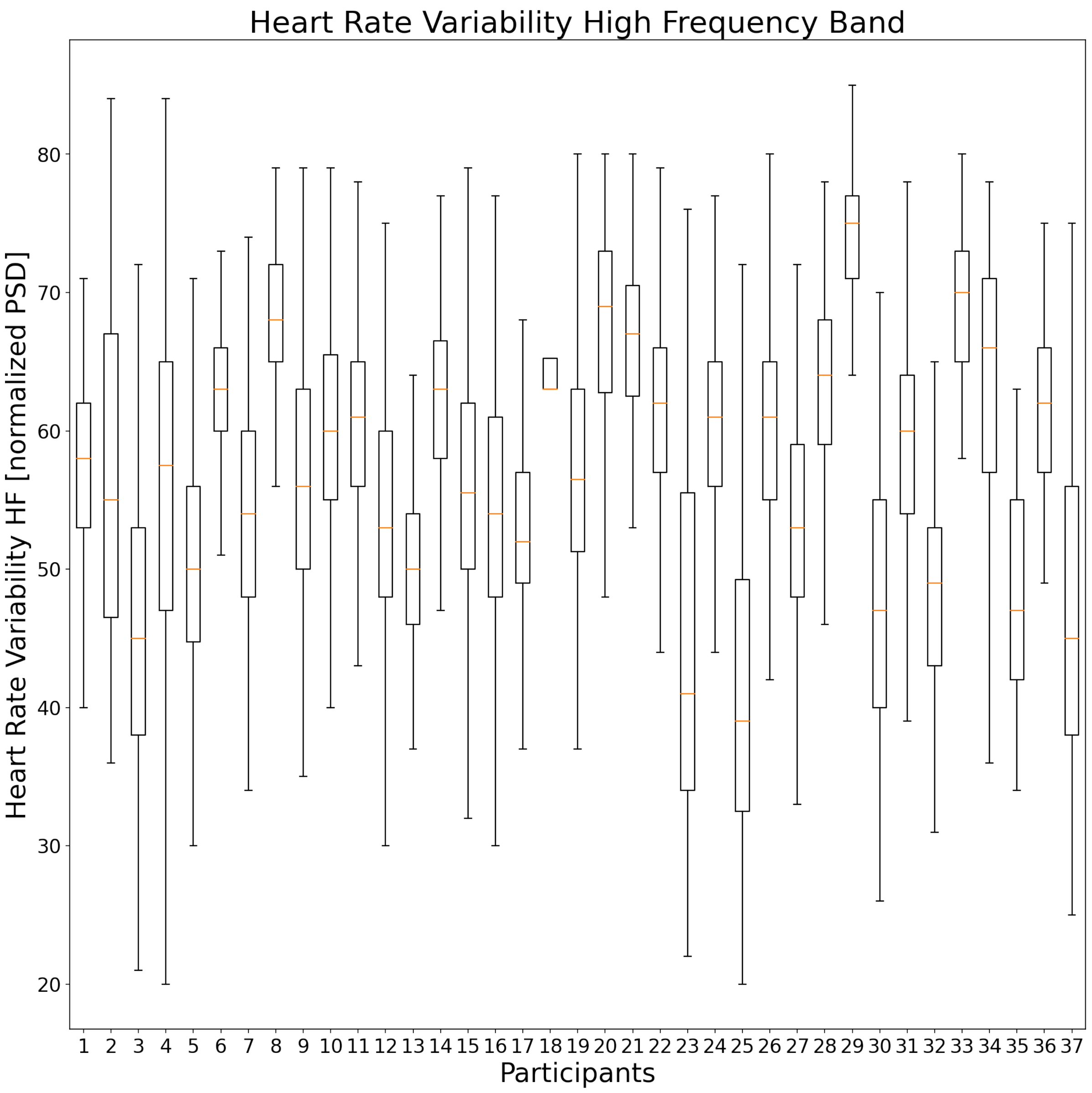

Heart Rate Variability High Frequency Band
Heart Rate Variability HF [normalized PSD]
20
30
40
50
60
70
80
1 2 3 4 5 6 7 8 9 10 11 12 13 14 15 16 17 18 19 20 21 22 23 24 25 26 27 28 29 30 31 32 33 34 35 36 37
Participants

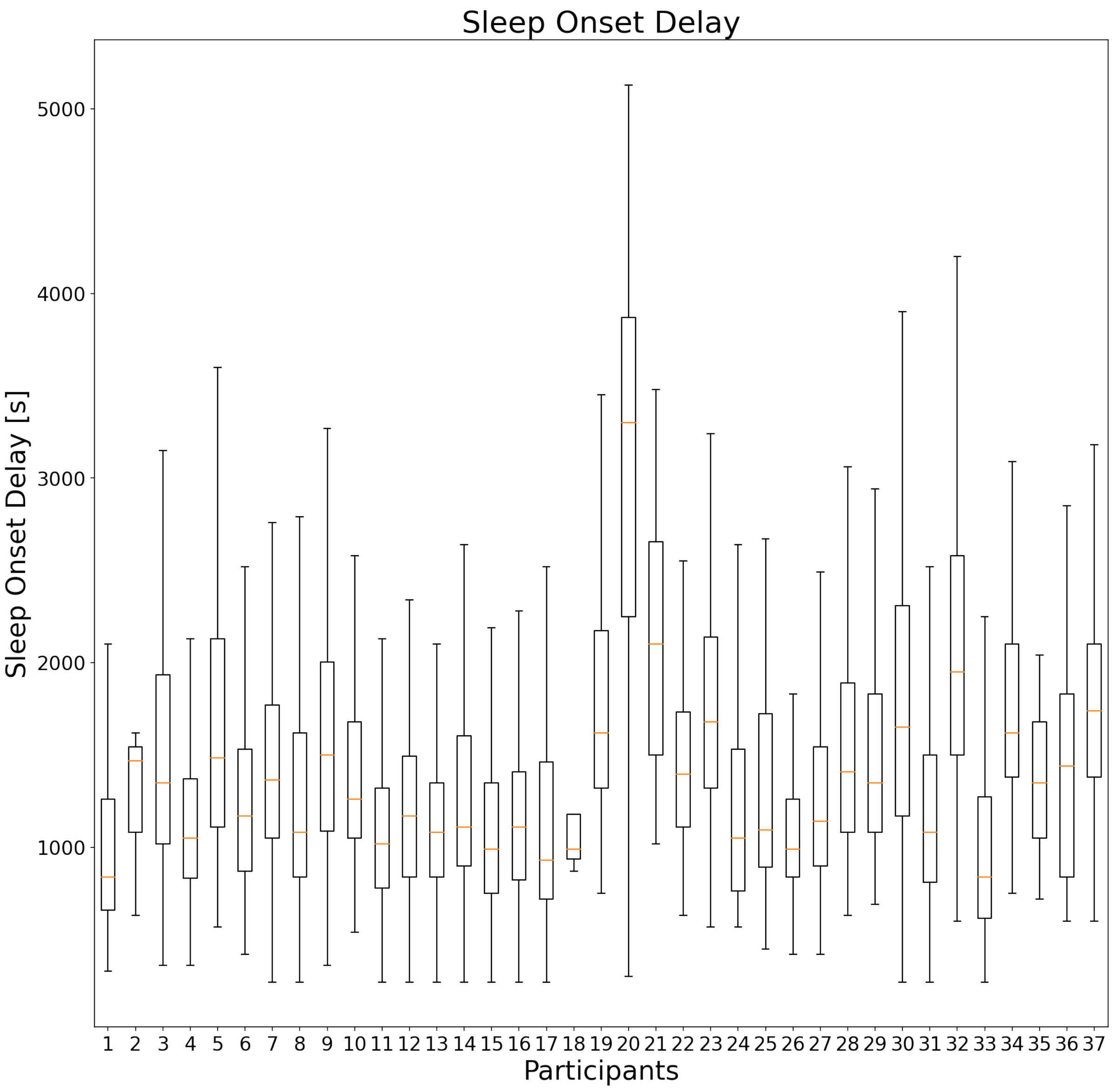

Sleep Onset Delay
Sleep Onset Delay [s]
5000
4000
3000
2000
1000
1 2 3 4 5 6 7 8 9 10 11 12 13 14 15 16 17 18 19 20 21 22 23 24 25 26 27 28 29 30 31 32 33 34 35 36 37
Participants

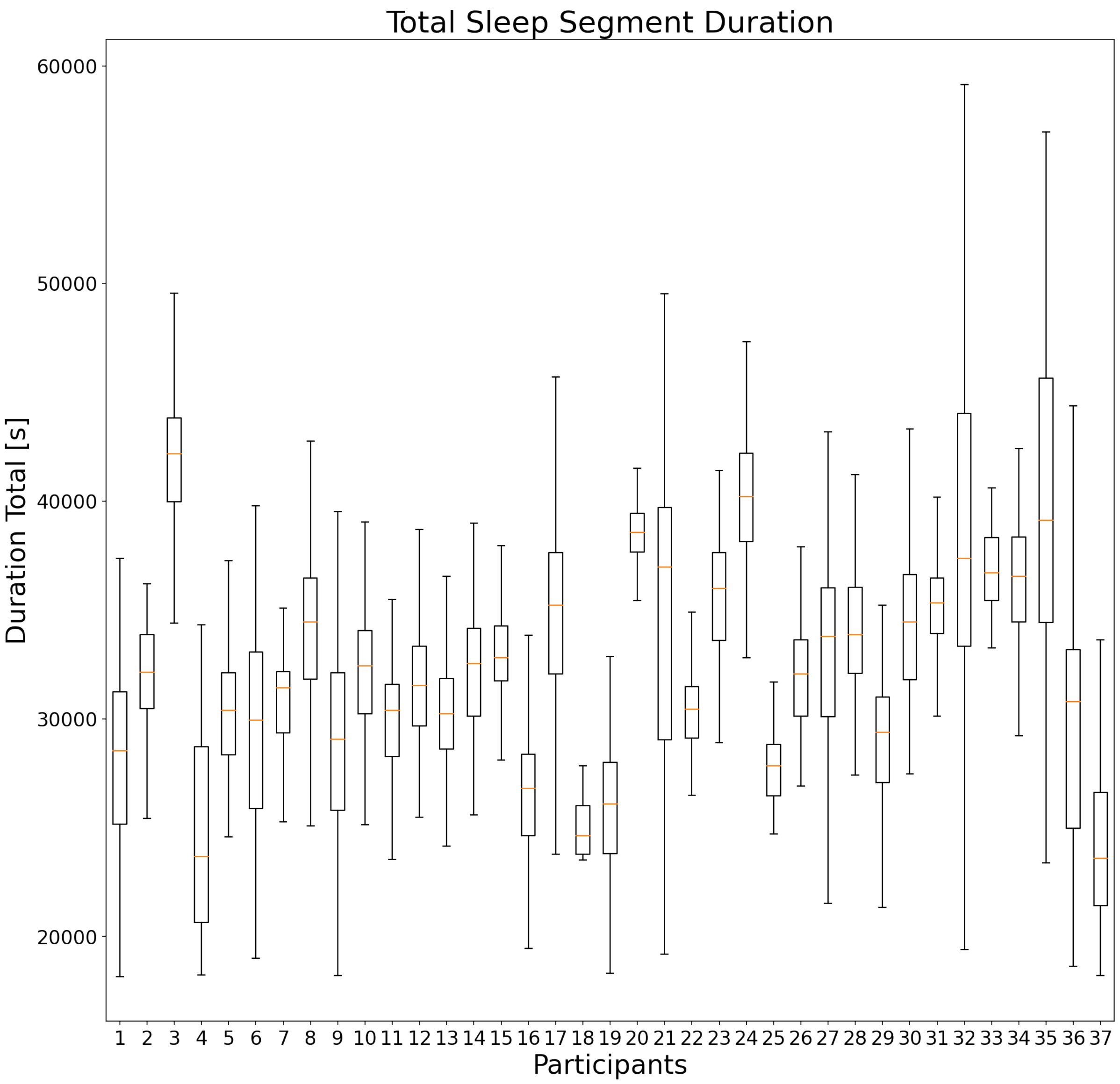

Total Sleep Segment Duration
Duration Total [s]
60000
50000
40000
30000
20000
1 2 3 4 5 6 7 8 9 10 11 12 13 14 15 16 17 18 19 20 21 22 23 24 25 26 27 28 29 30 31 32 33 34 35 36 37
Participants

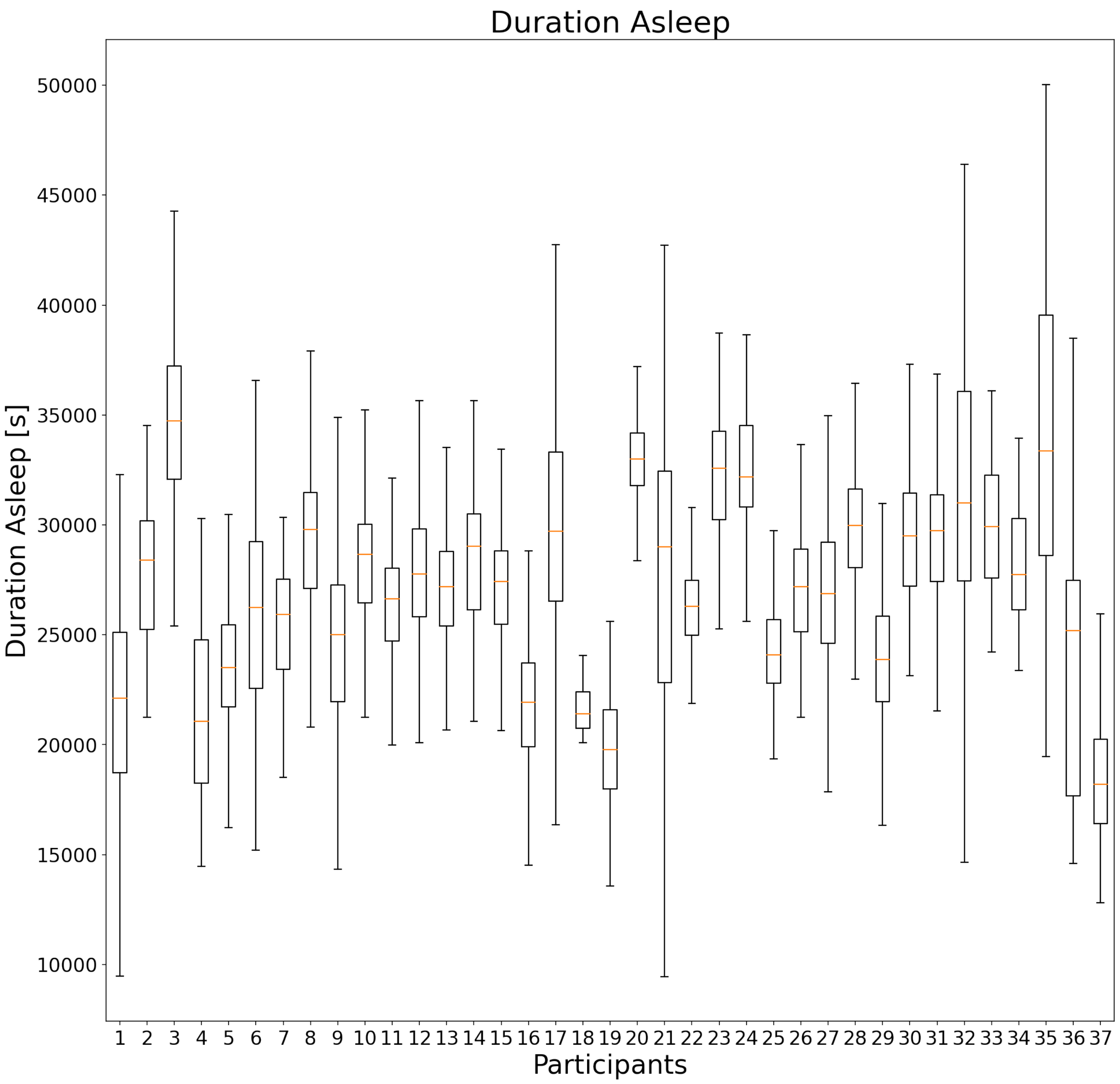

Duration Asleep
Duration Asleep [s]
50000
45000
40000
35000
30000
25000
20000
15000
10000
1 2 3 4 5 6 7 8 9 10 11 12 13 14 15 16 17 18 19 20 21 22 23 24 25 26 27 28 29 30 31 32 33 34 35 36 37
Participants

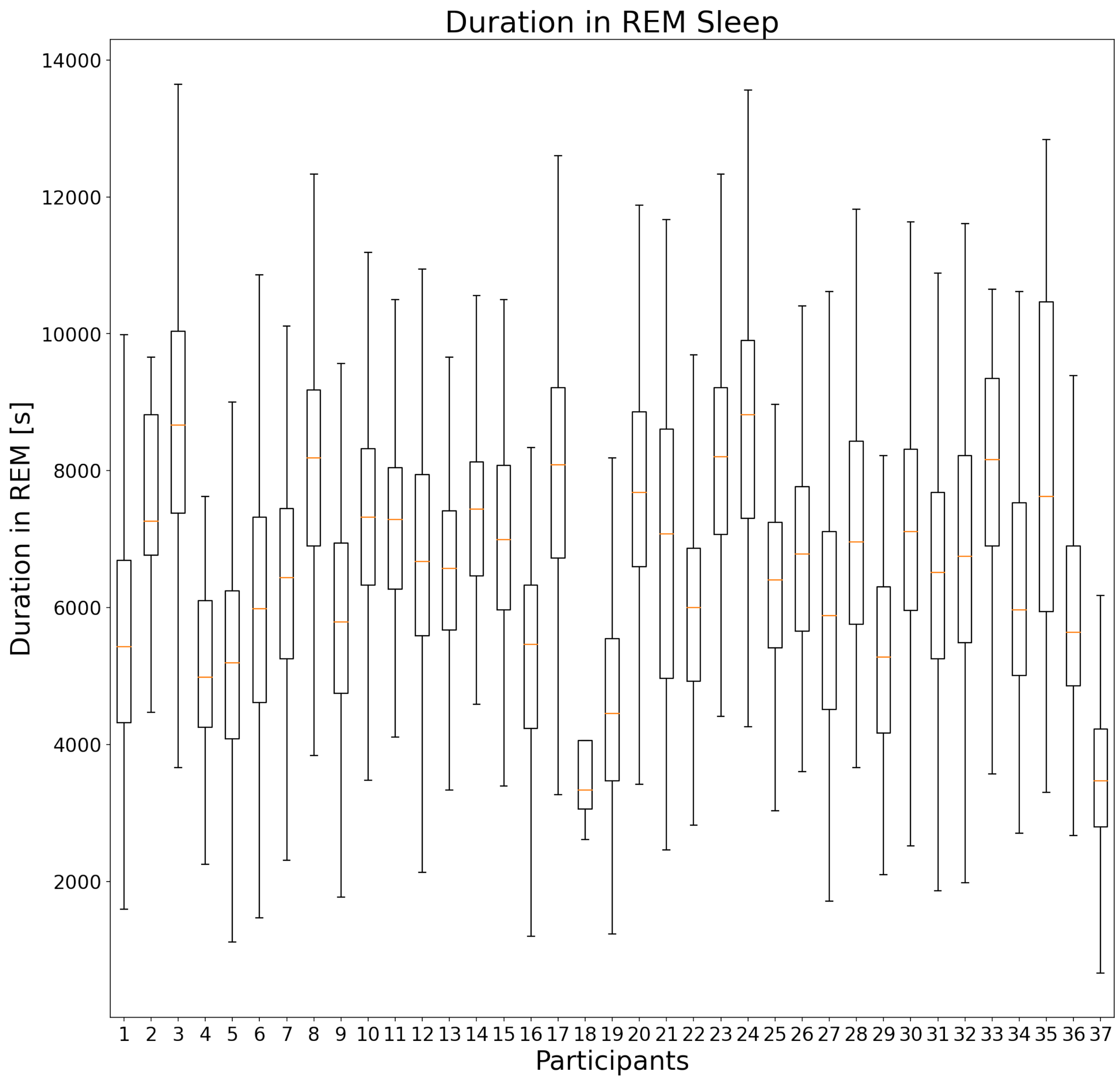
Duration in REM Sleep
Duration in REM [s]
14000
12000
10000
8000
6000
4000
2000
1 2 3 4 5 6 7 8 9 10 11 12 13 14 15 16 17 18 19 20 21 22 23 24 25 26 27 28 29 30 31 32 33 34 35 36 37
Participants

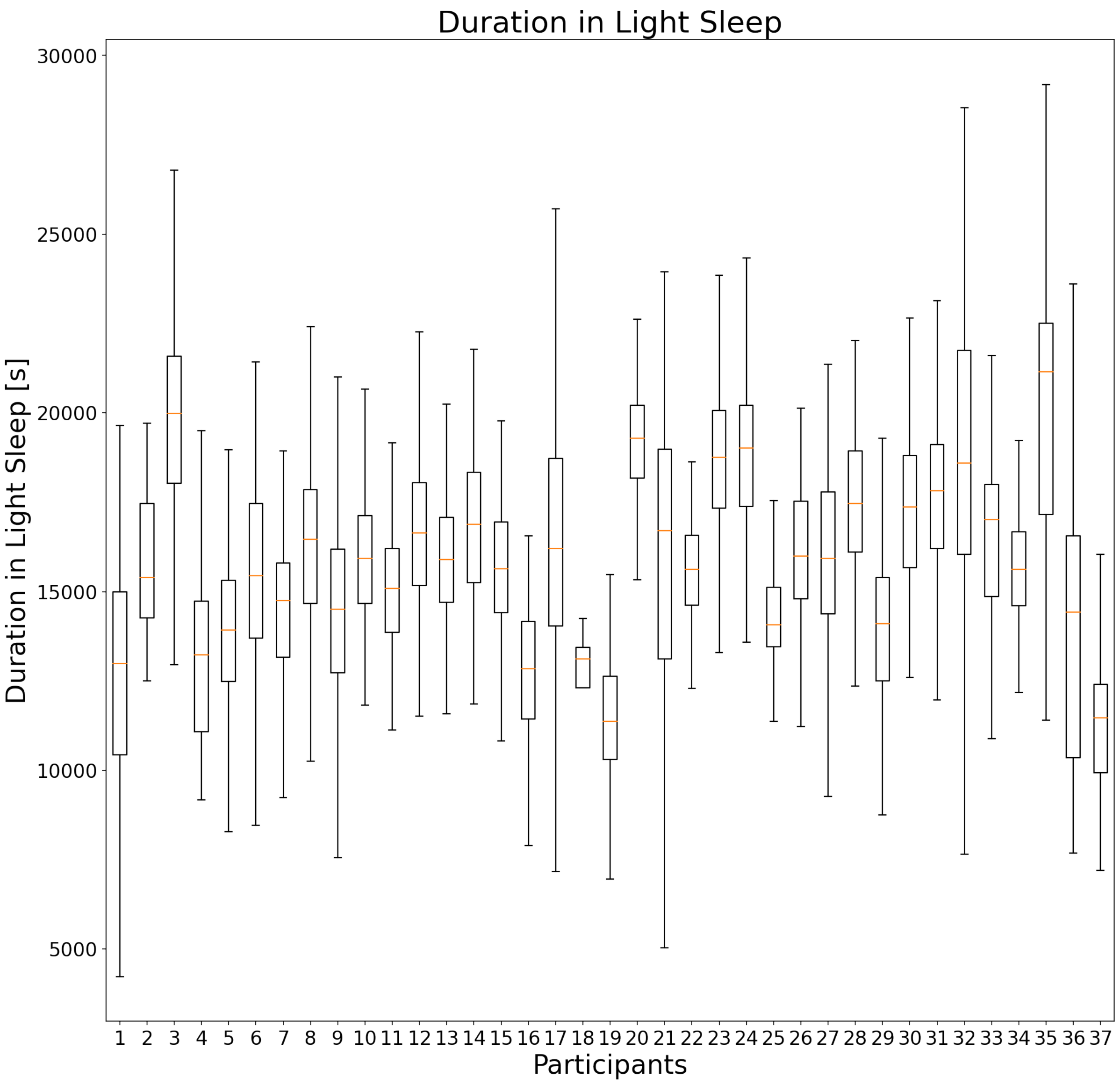
Duration in Light Sleep
Duration in Light Sleep [s]
30000
25000
20000
15000
10000
5000
1 2 3 4 5 6 7 8 9 10 11 12 13 14 15 16 17 18 19 20 21 22 23 24 25 26 27 28 29 30 31 32 33 34 35 36 37
Participants

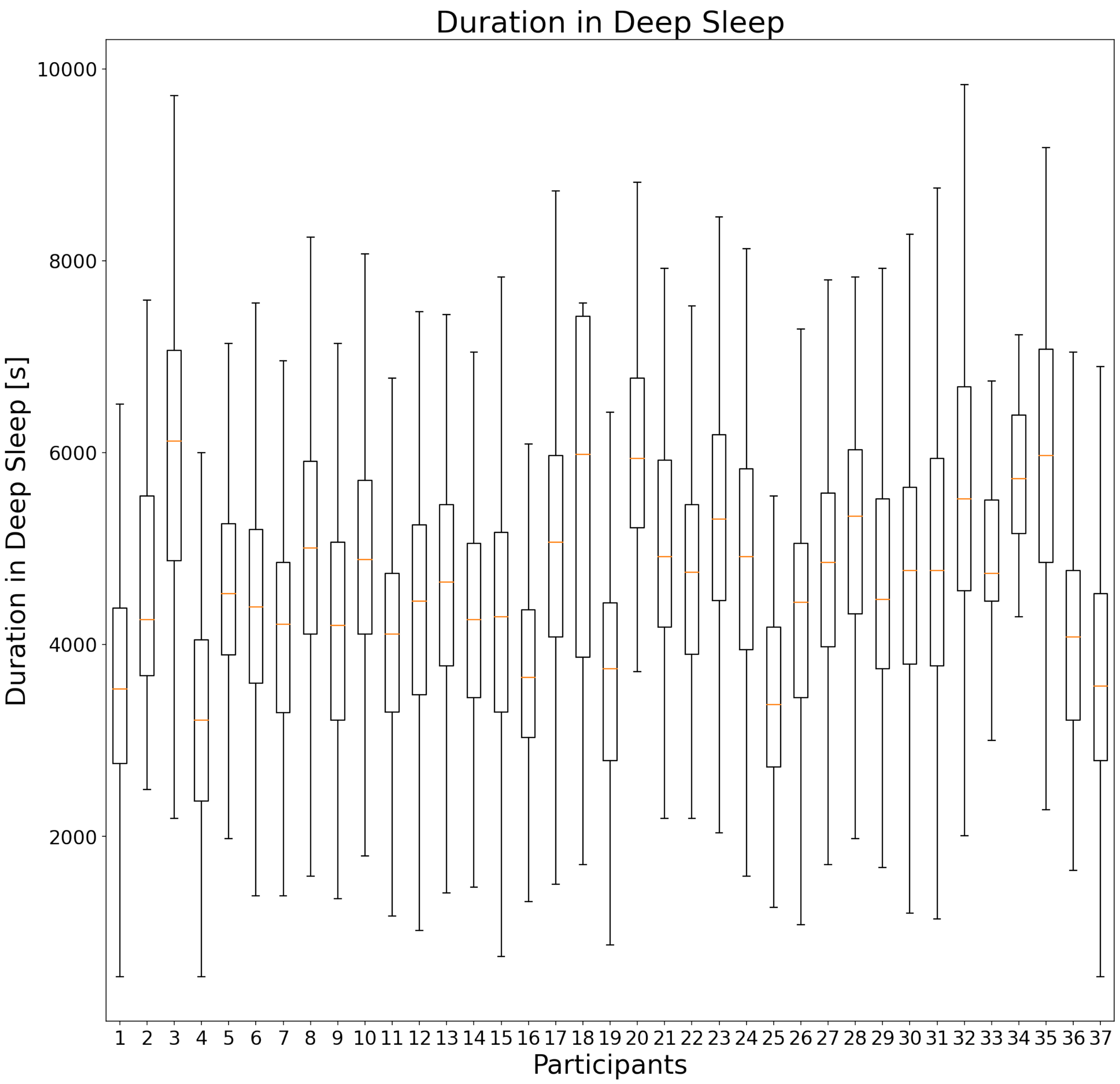
Duration in Deep Sleep
Duration in Deep Sleep [s]
10000
8000
6000
4000
2000
1 2 3 4 5 6 7 8 9 10 11 12 13 14 15 16 17 18 19 20 21 22 23 24 25 26 27 28 29 30 31 32 33 34 35 36 37
Participants

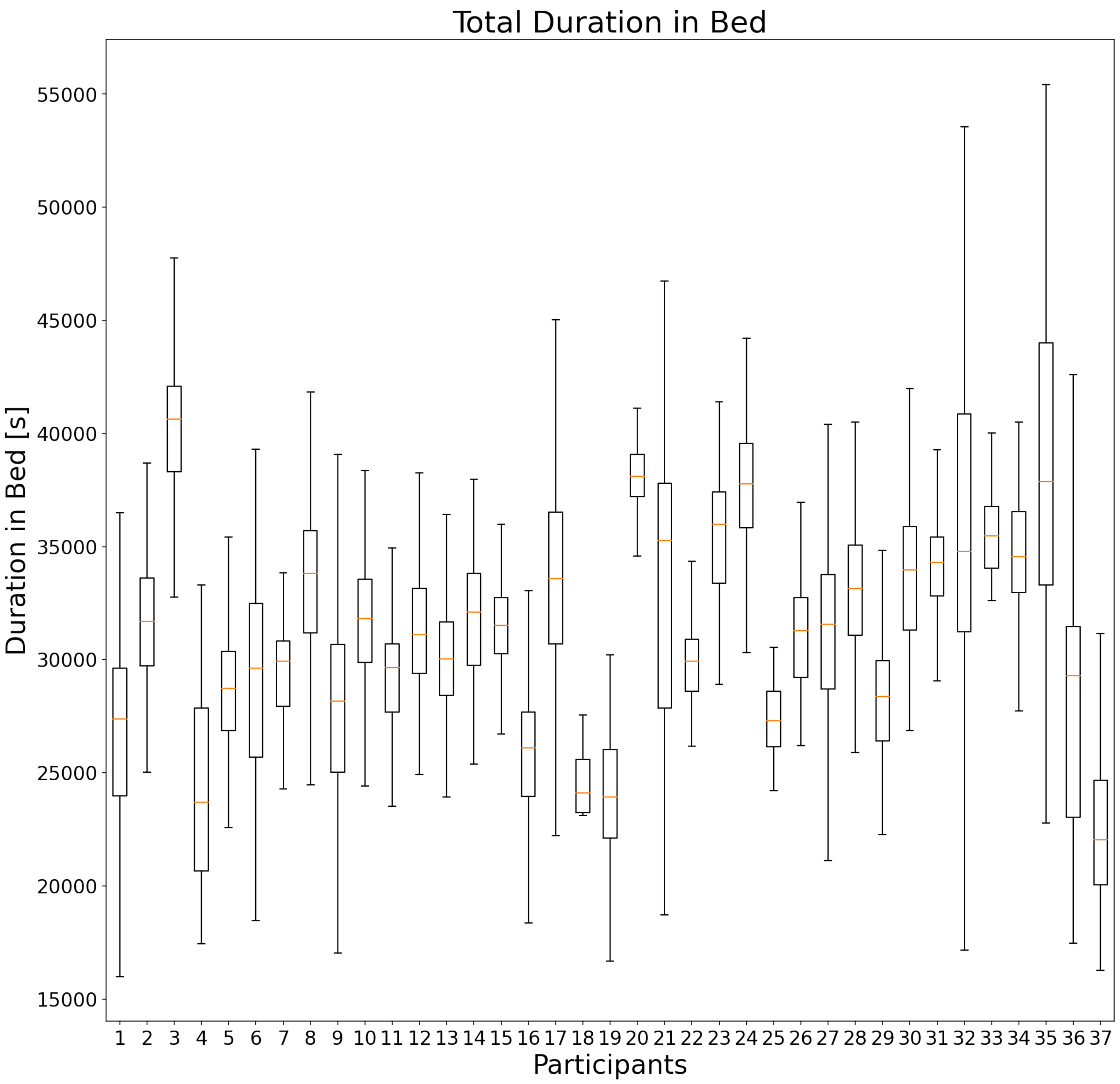

Total Duration in Bed
Duration in Bed [s]
55000
50000
45000
40000
35000
30000
25000
20000
15000
1 2 3 4 5 6 7 8 9 10 11 12 13 14 15 16 17 18 19 20 21 22 23 24 25 26 27 28 29 30 31 32 33 34 35 36 37
Participants

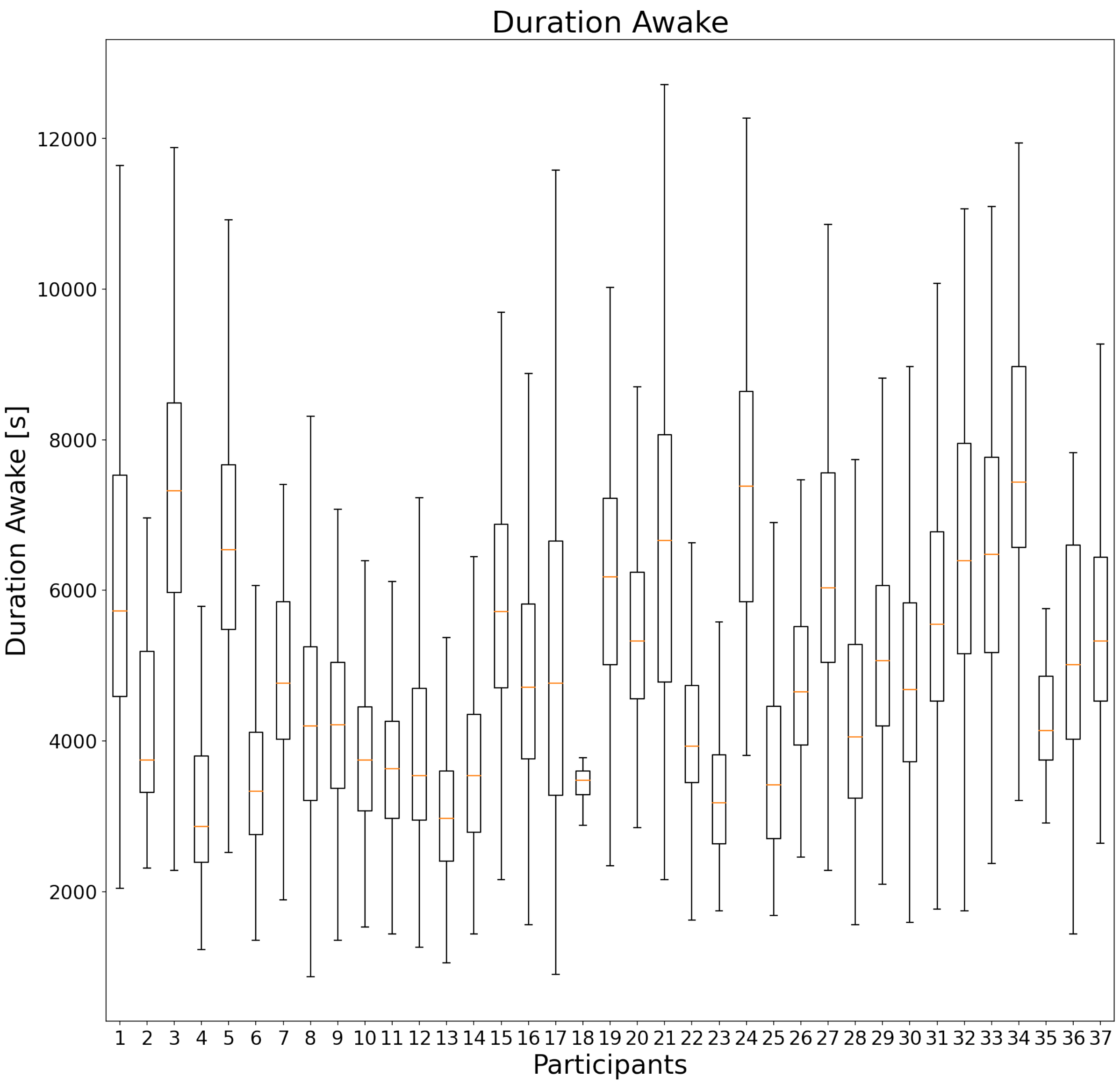

Duration Awake
Duration Awake [s]
12000
10000
8000
6000
4000
2000
1 2 3 4 5 6 7 8 9 10 11 12 13 14 15 16 17 18 19 20 21 22 23 24 25 26 27 28 29 30 31 32 33 34 35 36 37
Participants

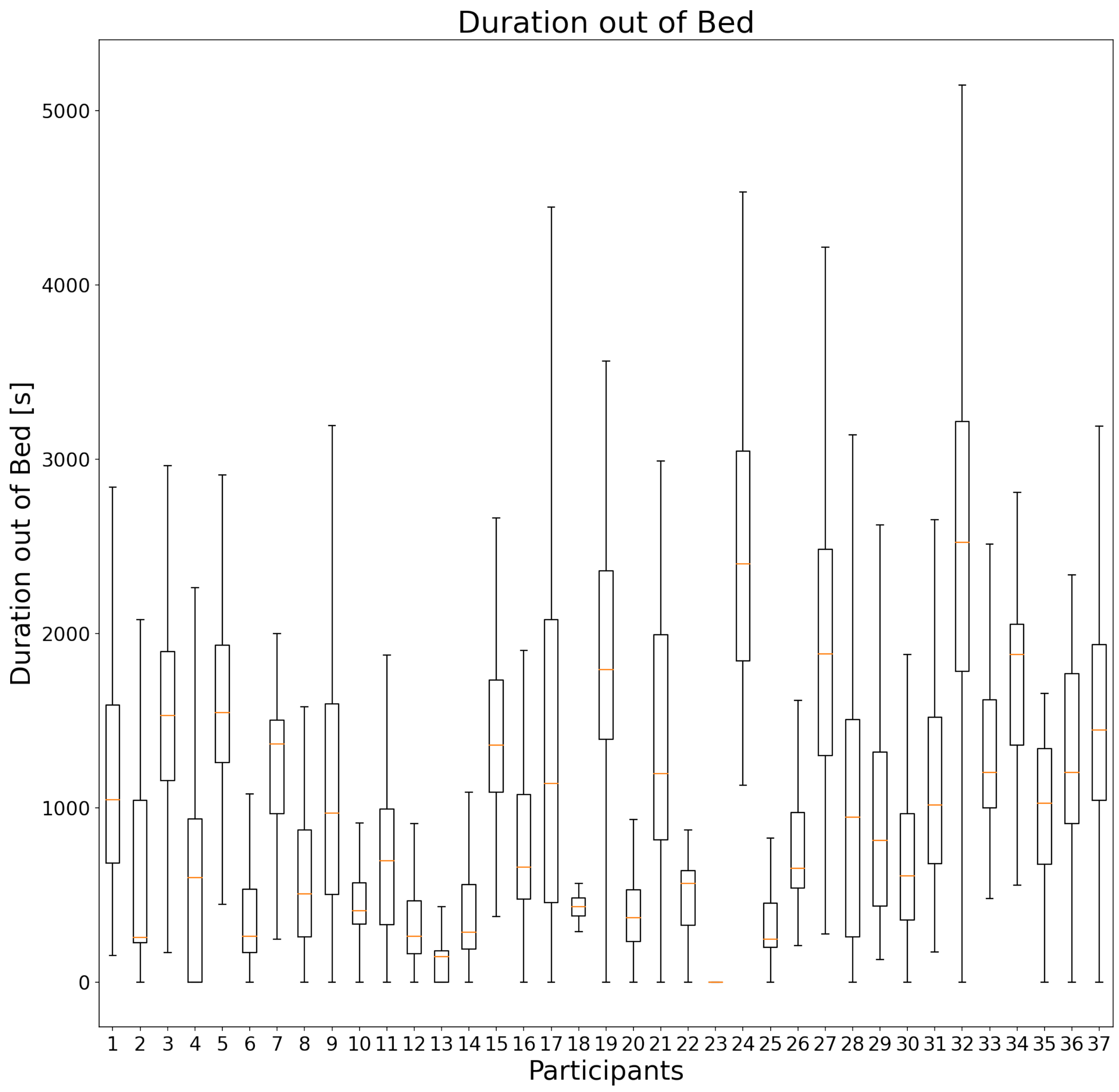

Duration out of Bed
Duration out of Bed [s]
0
1000
2000
3000
4000
5000
1 2 3 4 5 6 7 8 9 10 11 12 13 14 15 16 17 18 19 20 21 22 23 24 25 26 27 28 29 30 31 32 33 34 35 36 37
Participants

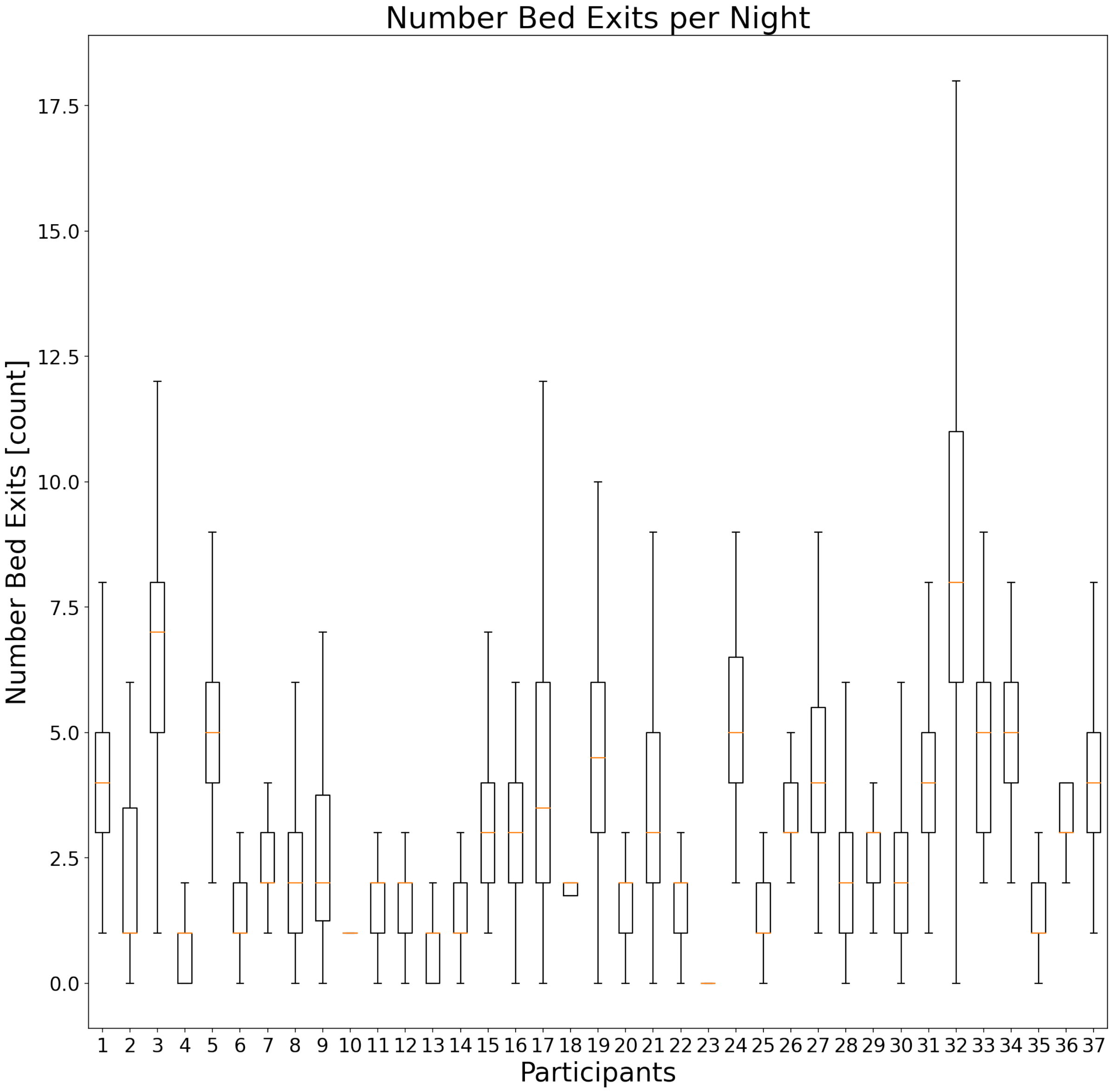

Number Bed Exits per Night
Number Bed Exits [count]
0.0
2.5
5.0
7.5
10.0
12.5
15.0
17.5
Participants
1 2 3 4 5 6 7 8 9 10 11 12 13 14 15 16 17 18 19 20 21 22 23 24 25 26 27 28 29 30 31 32 33 34 35 36 37

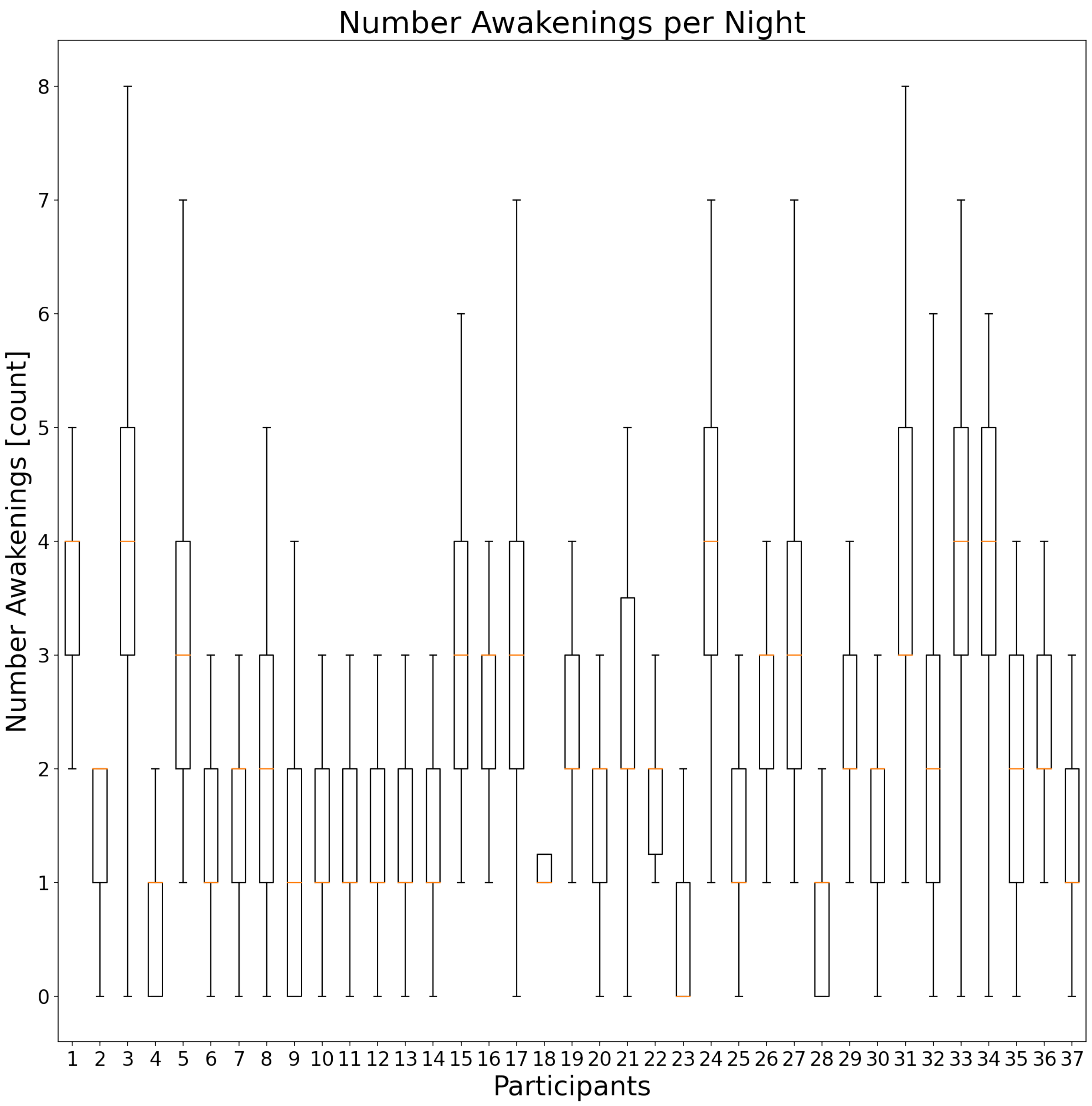
Number Awakenings per Night
Number Awakenings [count]
0
1
2
3
4
5
6
7
8
1 2 3 4 5 6 7 8 9 10 11 12 13 14 15 16 17 18 19 20 21 22 23 24 25 26 27 28 29 30 31 32 33 34 35 36 37
Participants

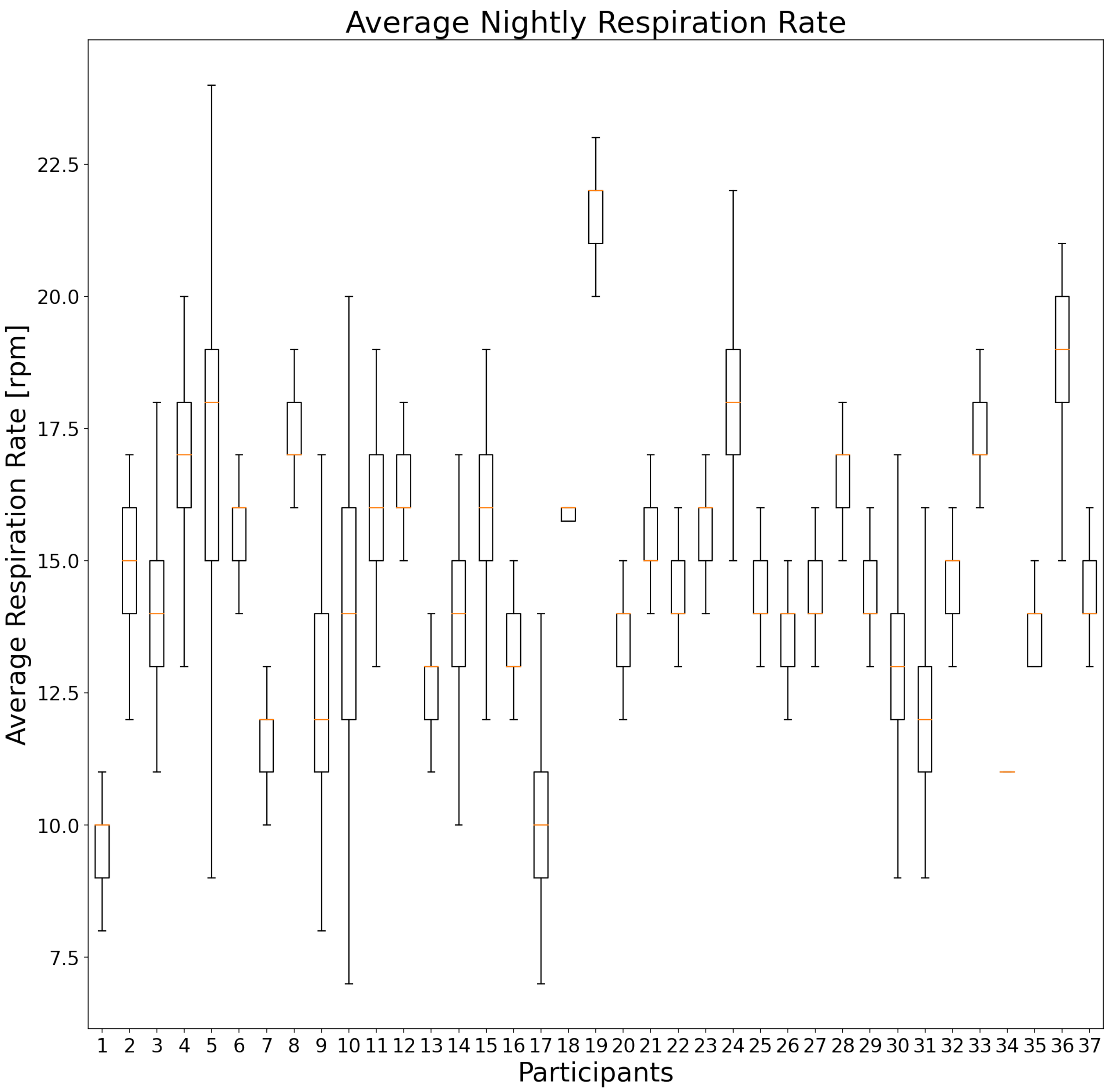
Average Nightly Respiration Rate
Average Respiration Rate [rpm]
22.5
20.0
17.5
15.0
12.5
10.0
7.5
1 2 3 4 5 6 7 8 9 10 11 12 13 14 15 16 17 18 19 20 21 22 23 24 25 26 27 28 29 30 31 32 33 34 35 36 37
Participants
